# Vitamin D—A prominent immunomodulator to prevent COVID‐19 infection

**DOI:** 10.1111/1756-185X.14477

**Published:** 2022-10-29

**Authors:** Sumel Ashique, Kirti Gupta, Gaurav Gupta, Neeraj Mishra, Sachin Kumar Singh, Sheetu Wadhwa, Monica Gulati, Harish Dureja, Flavia Zacconi, Brian G. Oliver, Keshav Raj Paudel, Philip M. Hansbro, Dinesh Kumar Chellappan, Kamal Dua

**Affiliations:** ^1^ Department of Pharmaceutics Bharat Institute of Technology (BIT), School of Pharmacy Meerut India; ^2^ Department of Pharmacology, MM College of Pharmacy Maharishi Markandeshwar (Deemed to be) University Ambala India; ^3^ School of Pharmacy Suresh Gyan Vihar University Jaipur India; ^4^ Department of Pharmacology, Saveetha Dental College, Saveetha Institute of Medical and Technical Sciences Saveetha University Chennai India; ^5^ Uttaranchal Institute of Pharmaceutical Sciences Uttaranchal University Dehradun India; ^6^ Department of Pharmaceutics, Amity Institute of Pharmacy Amity University Madhya Pradesh (AUMP) Gwalior India; ^7^ School of Pharmaceutical Sciences Lovely Professional University Jalandhar India; ^8^ Faculty of Health, Australian Research Centre in Complementary and Integrative Medicine University of Technology Sydney New South Wales Ultimo Australia; ^9^ Department of Pharmaceutical Sciences Maharshi Dayanand University Rohtak India; ^10^ Facultad de Química y de Farmacia Pontificia Universidad Católica de Chile Santiago Chile; ^11^ Institute for Biological and Medical Engineering, Schools of Engineering, Medicine and Biological Sciences Pontificia Universidad Católica de Chile Santiago Chile; ^12^ Woolcock Institute of Medical Research University of Sydney New South Wales Sydney Australia; ^13^ School of Life Sciences, Faculty of Science University of Technology Sydney 2007 New South Wales Sydney Australia; ^14^ Centre for Inflammation Centenary Institute and University of Technology Sydney, Faculty of Science, School of Life Sciences New South Wales Sydney Australia; ^15^ Department of Life Sciences, School of Pharmacy International Medical University Kuala Lumpur Malaysia; ^16^ Discipline of Pharmacy, Graduate School of Health University of Technology Sydney New South Wales Sydney Australia

**Keywords:** doses, immune system, mechanism, severe acute respiratory syndrome coronavirus 2, vitamin D

## Abstract

COVID‐19 remains a life‐threatening infectious disease worldwide. Several bio‐active agents have been tested and evaluated in an effort to contain this disease. Unfortunately, none of the therapies have been successful, owing to their safety concerns and the presence of various adverse effects. Various countries have developed vaccines as a preventive measure; however, they have not been widely accepted as effective strategies. The virus has proven to be exceedingly contagious and lethal, so finding an effective treatment strategy has been a top priority in medical research. The significance of vitamin D in influencing many components of the innate and adaptive immune systems is examined in this study. This review aims to summarize the research on the use of vitamin D for COVID‐19 treatment and prevention. Vitamin D supplementation has now become an efficient option to boost the immune response for all ages in preventing the spread of infection. Vitamin D is an immunomodulator that treats infected lung tissue by improving innate and adaptive immune responses and downregulating the inflammatory cascades. The preventive action exerted by vitamin D supplementation (at a specific dose) has been accepted by several observational research investigations and clinical trials on the avoidance of viral and acute respiratory dysfunctions. To assess the existing consensus about vitamin D supplementation as a strategy to treat and prevent the development and progression of COVID‐19 disease, this review intends to synthesize the evidence around vitamin D in relation to COVID‐19 infection.

## 1 INTRODUCTION

Genome homology of reports that severe acute respiratory syndrome coronavirus 2 (SARS‐CoV‐2) uses the cell receptors of dipeptidyl peptidase and angiotensin‐converting enzyme 2 (ACE2) to gain entry into the host cell.^1^ Similar to severe acute respiratory syndrome coronavirus (SARS‐CoV) and Middle‐East respiratory syndrome coronavirus (MERS‐CoV), the novel virus SARS‐CoV‐2 also causes inflammation and releases pro‐inflammatory mediators, such as tumor necrosis factor‐α (TNF‐α) and interleukin‐1β (IL‐1 β) and IL‐6.^2^ Both innate and adaptive immunity play a significant role in eliciting immediate protective immune responses. Several studies have revealed the significant role of vitamin D in controlling these innate as well as adaptive immune responses concurrently via vitamin D receptor (VDR) localized in immune cells, including T cells (CD4^+^ and CD8^+^), B cells, monocytes, neutrophils, macrophages, and dendritic cells.^3^ Individuals who lacked vitamin D had upregulated expression of IL‐6, where the TNF‐α was found to activate phenotypes of monocytes.^4^ Host responses are occasionally altered as the result of overexposure to inflammatory mediators, and the apparent “cytokine storm” causes critical complications in SARS‐CoV‐2‐infected patients like those in acute respiratory distress syndrome (ARDS).^5^ Immune defenses can be boosted by vitamin D supplementation, which balances inflammation versus anti‐inflammation. Persistent lack of vitamin D is one of the increasing pathological states worldwide, affecting more than 1 billion of individuals.^6^ Research has revealed a prospective relationship between insufficient vitamin D and the occurrence of systemic infection along with several other diseases.^7^, ^8^, ^9^ Vitamin D deficiency alters the body's immunity because vitamin D is a key player in immune modulation, as it affects secretion of antiviral peptides and hence improves innate immunity, which is responsible for maintaining mucosal integrity and defense mechanisms.^10^, ^11^, ^12^ The current hypothesis states that a lack of vitamin D could reduce respiratory immune responses and enhance the chances of SARS‐CoV‐2 infection severity and mortality rate.^13^, ^14^ Along with this hypothesis, numerous retrospective studies have represented the interrelationship between vitamin D content and the severity of SARS‐CoV‐2 infection.^15^, ^16^, ^17^, ^18^, ^19^, ^20^ As a result, enhancing the immune system by administering vitamin D has become a significant factor. However, there are limited data concerning vitamin D and its ability to successfully prevent the infection; there is a need for more research about vitamin D supplementation and how it can be useful to enhance the immune system and prevent further spread of SARS‐CoV‐2. Evidence from past literature has illustrated the importance of vitamin D in the chronic phase of diseases leading to high mortality rates in patients with coronavirus disease 2019 (COVID‐19). Immune responses are well‐documented as being modulated by vitamin D. There has been a much interest in vitamin D's potential to reduce or avoid negative immunological reactions caused by the severe effects of COVID‐19 on the immune system. A narrative synthesis is necessary to summarize the current level of knowledge in this topic because several new studies have just been released.^21^ In order to assess the existing consensus about vitamin D supplementation as a strategy to treat and/or prevent the development or progression of COVID‐19, this review intends to synthesize the evidence around vitamin D in relation to COVID‐19. With reference to significant research and systematic reviews that have been published, we provide an overview of the current level of knowledge in this field. This review was prepared by collecting the relevant scientific information from PubMed, Web of Science/Scopus, and Google Scholar using the following phrases in all possible combinations: “role of vitamin D in respiratory diseases” (292 results), “role of vitamin D in covid‐19” (9 results), “molecular mechanisms of vitamin D" (12 results), “role of vitamin D in viral disease” (5 results). This survey identified a total of 318 published articles, but after scrutiny only 42 included original research evidence focused on the involvement of vitamin D in COVID‐19, published between 2019 and 2022. The selected studies tested the role of vitamin D in diverse respiratory dysfunctions including in vitro and ex vivo findings. Evidence pertaining to the impact of vitamin D in comorbidities, other organ injuries, and other viral manifestations were excluded from the present review.

## 2 POTENTIAL MOLECULAR MECHANISM OF VITAMIN D

**FIGURE 1:**
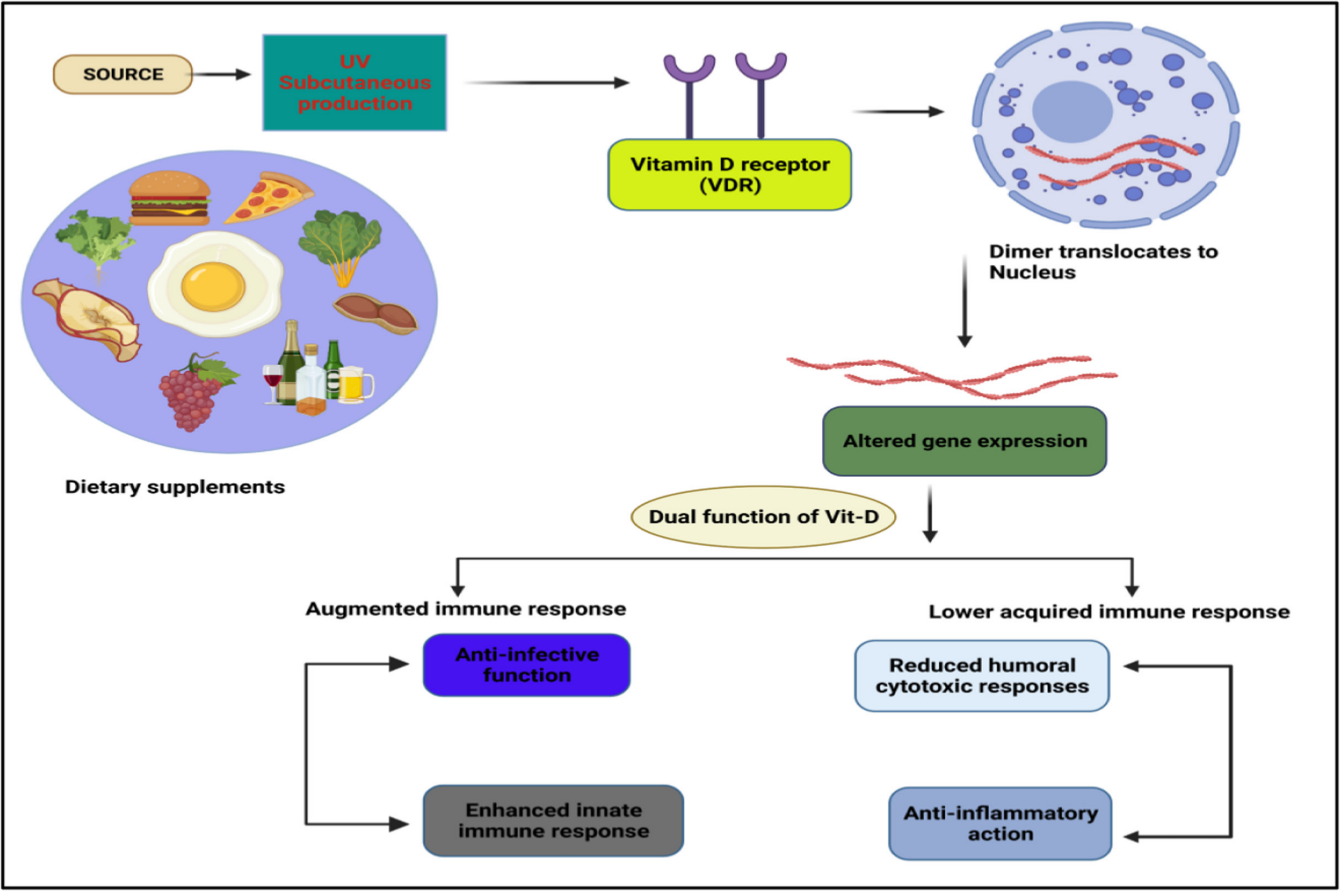
Representation of dual role of vitamin D on the immune system and inflammation. Vitamin D has the potential to alter expression of several genes, showing increased innate immune response and reduced acquired immune response. UV, ultraviolet rays; VDR, vitamin D receptor

The three main mechanisms involved in reducing microbial infection are physically oriented barriers, cellular immunity, and humoral immunity.^22^ After administration, the binding of vitamin D to its receptors in the cell causes the activation of retinoid X receptors, resulting in the relocation of the vitamin D receptor element. The VDR controls the presence of several host genes, such as those for β‐defensin and cathelicidin.^23^ Innate immunity of the cell is also enhanced through vitamin D by upregulation of human cathelicidin LL‐37, 1,25‐di‐hydroxy vitamin D, and defensins, and continuing tight junctions, adherens junctions, and gap junctions.^24^, ^25^, ^26^, ^27^ Cathelicidins have direct antipathogenic activity in response to a broad range of microorganisms, particularly bacteria (Gram‐positive/Gram‐negative), viruses (enveloped/non‐enveloped), and fungi.^28^ The several functions performed by cathelicidin include promoting the triggering of inflammatory cascades and prompting chemotaxis of leukocytes such as monocytes, neutrophils, macrophages, and T lymphocytes. This further helps to clear pathogens from the respiratory tract by persuading apoptotic and autophagic events in epithelial cells exposed to the virus.^10^, ^29^ Vitamin D also impacts Toll‐like receptors, which play a significant role in the body's innate immune response because they recognize harmful proteins.^30^ Vitamin D regulates several genes, such as defensins, which directly destroy virus membrane. When activated, VDR attaches to the VDR element of the cathelicidin promoter region, triggering host defense against viral infections. The release of nitric oxide is also enhanced by vitamin D, which helps in mediating innate immunity. This is exhibited primarily by dampening T‐cell proliferation and the resulting conversion of T helper type 1 (Th1) cells into Th2 cells.^31^, ^32^ Vitamin D also impacts the maturation of T cells and can further shift Th17 cells toward regulatory T cells, which are anti‐inflammatory. By following this mechanism, vitamin D downregulates pro‐inflammatory cytokines such as IL‐1, IL‐6, IL‐12, and TNF‐α.^33^, ^34^, ^35^ Gene expression associated with anti‐oxidation is improved through vitamin D intake; as a result, glutathione production is enhanced, which shows antimicrobial activities,^36^, ^37^, ^38^ regulatory T cell‐improving features of vitamin D are resolved either by direct or indirect signaling pathways that also influence tolerogenic dendritic cells.^39^ Dendritic cells play a key role in vitamin D‐moderated immunoregulation.^40^ In addition, vitamin D directly impedes the nuclear factor‐κB pathway, therefore lowering the generation of pro‐inflammatory mediators. Apparently, the control of cytokine regulation and differentiation of T cells shows the dual role of vitamin D in immunopathological conditions.^41^ VDR and CYP27B1 may be significant target tissues for vitamin D in the endocrine system. Several studies have reported the downregulation of ACE2 receptors by vitamin D, hence preventing the manifestations of COVID‐19.^42^ Figure 1 illustrates how vitamin D can boost the immune response.

## 3 INTERPLAY BETWEEN VITAMIN D AND ACE2 RECEPTOR

**FIGURE 2:**
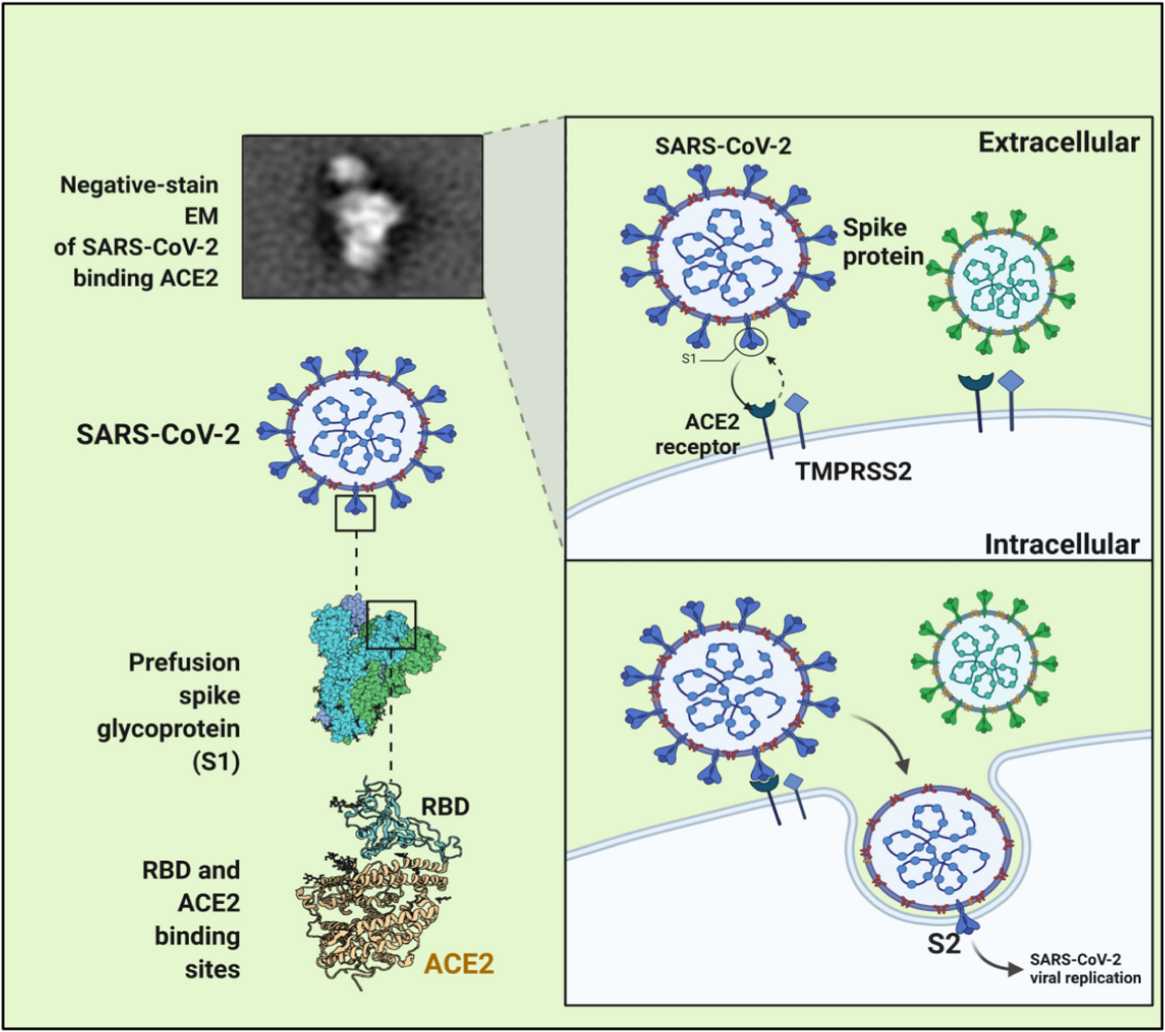
Entry mechanism of severe acute respiratory syndrome coronavirus 2 (SARS‐CoV‐2) through host angiotensin‐converting enzyme 2 (ACE2). SARS‐CoV‐2 binds to the surface of host cells via several receptors (ACE2, neuropilin‐1, AXL, and antibody‐Fcγ receptor complexes). The spike (S) protein goes through conformational transition from prefusion to postfusion by using proteases (furin, TMPRSS2, and cathepsins). SARS‐CoV‐2 attaches with the cell receptor ACE2, which comprises integral membrane protein, and moves to the surface of cells after transcription with its N‐terminal signal peptide and binds by C‐terminal transmembrane domain. When receptor‐binding domain starts binding with the tips of one lobe of ACE2 the viral entry initiates

**FIGURE 3:**
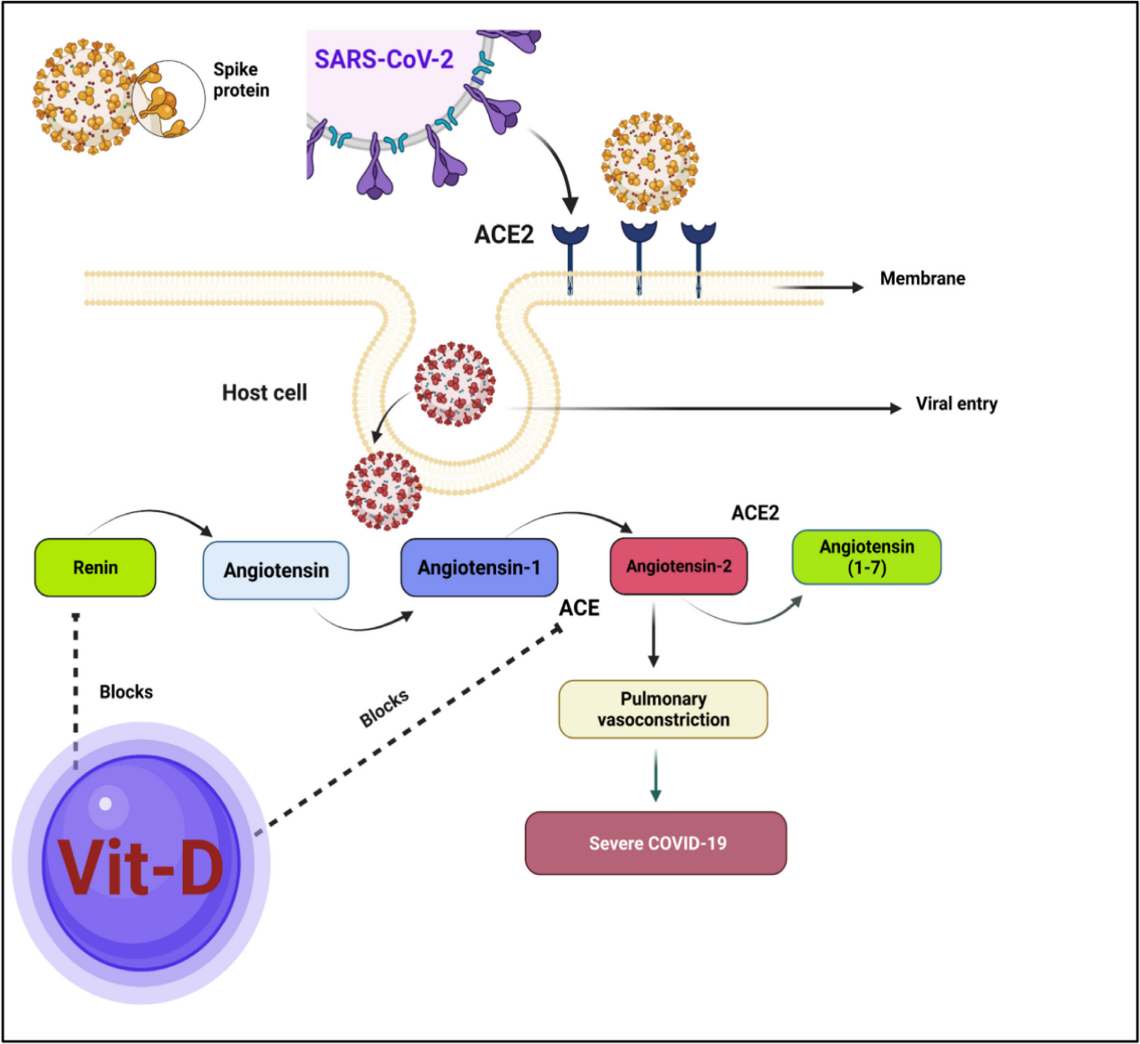
Vitamin D functions in the severe acute respiratory syndrome coronavirus 2 (SARS‐CoV‐2) response after angiotensin‐converting enzyme (ACE). The ACE converts Ang I into Ang II. Vasoconstriction, inflammation, and apoptosis are caused by Ang II binding to the Ang II type 1 receptor. Ang (1‐7) counteracts Ang II's effects. As a result, the equilibrium between ACE and ACE2 levels affects the endogenous content of Ang II and Ang (1‐7) 8. Additionally, vitamin D also reduces RAS activity by diminishing renin

Acute respiratory distress syndrome is mainly due to endothelial defects and enhanced barrier permeability, resulting in increased concurrent diseases and death rates in SARS‐CoV‐2‐infected patients. The nutrition D‐mediated pathway might also keep away the risk of mild respiratory dysfunction. In addition, 1,25(OH)_2_ D3 is also responsible for suppressing renin, ACE, and angiotensin II (Ang II) expression and for increasing ACE‐2 in lipopolysaccharide‐mediated acute respiratory dysfunction. Nutrition D decreased lipopolysaccharide‐triggered lung injury by various means, including by partially prompting ACE2 and Ang (1‐7) axis action, resulting in the suppression of renin and the ACE and Ang II or angiotensin II receptor type 1 events and drastic exposure of VDR in the lungs. It was concluded that the deactivation of VDR led to dyshomeostasis in the renin‐angiotensin system. VDR and 1,25(OH)_2_ D are also found to attenuate fibrotic cascades in kidneys, lungs, and liver through negative feedback of the renin‐angiotensin system and the hindering of nuclear factor‐κB.^43^ Besides mediating these protective functions, VDR also evades sepsis‐associated lung damage by interfering with the angiopoietin‐2‐TEK receptor triggering tyrosine kinase‐mediated myosin light‐chain kinase events.^44^ Vitamin D administration diminishes the acute lung injury by abolishing various pro‐inflammatory actions, inhibiting lung epithelium‐endothelium barrier transfer, and adjusting the renin‐angiotensin system. The analog of vitamin D (Paricalcitol) prevents alveolar barrier function, alleviating lipopolysaccharide‐linked acute lung injury. Figure 2 depicts the entry mechanism of SARS‐CoV‐2 through host ACE2, and Figure 3 shows the impact of vitamin D on ACE2 inhibitors.

## 4 DOES VITAMIN D EXERT ANTI‐VIRAL POTENTIAL?

Several research and review studies have investigated if vitamin D can lower the chances of microbial infections.^45^ A current review also described the active contribution of vitamin D supplementation in patients with COVID‐19, thereby reducing the mortality rate.^46^ As observed in several rodent and human cell lines, lymphopenia is a common and significant complication in critically affected SARS‐CoV‐2 patients. Vitamin D can provide a protective mechanism for experimental interstitial pneumonitis.^47^ Vitamin D therapy has been proven in a number of in vitro experiments to play a crucial role in maintaining local respiratory homeostasis, mainly via dual pathways: stimulation of the expression of antimicrobial peptides or by direct proliferation of the respiratory infection causing virus.^48^ Several investigations have found that vitamin D has antiviral properties, mostly against enveloped viruses. In respiratory viral infections, vitamin D regulates the immune system by raising the number of CD8^+^ T cells that are virus‐specific (such as against influenza and Epstein‐Barr viruses). Vitamin D also enhances macrophage development and oxidative burst capability while decreasing autophagy (linked to viral replication).

## 5 THE LACK OF VITAMIN D IN COVID‐19 PATIENTS

Lips and co‐workers report that vitamin D levels are continuing to rise in the populations of around 40 nations. However, more than 50% deficiency is reported among elderly patients. Vitamin D deficiency is found worldwide and is a major undiagnosed and untreated dietary deficiency (with more than 1 billion people being deficient).^49^ It was reported that human dipeptidyl peptidase‐4/CD26 has a link between the S_1_ domains of the SARS‐CoV‐2 spike glycoprotein, which may act as an ideal virulence feature in this infection.^50^ This appearance of the dipeptidyl peptidase‐4/CD26 receptor can be minimized by administering careful vitamin D supplementation. It was also stated that adjusting vitamin D supplementation could decrease the adverse downstream immunological sequelae, such as an increase in IL‐6, and late interferon‐γ response in the infected victims. A recent study demonstrated the direct association between vitamin D scarcity and the SARS‐CoV‐2‐infected population.^51^ It showed that those who were given the most vitamin D were at lower risk for SARS‐CoV‐2 infection. An additional study found that 84.6% of critically ill COVID‐19 patients in intensive care units (ICUs) were vitamin D deficient (12 ng/mL), compared with only 57.1% of patients in medical wards.^52^ Also, COVID‐19 patients in ICUs had lower 25(OH)D serum concentrations than non‐ICU patients, verified in a study of 34 SARS‐CoV‐2‐infected patients.^53^ In addition, another study found a link between noninvasive breathing assistance and dependence unit admission (*P* = 0.042), with older affected individuals having worse consequences.^54^ Several studies have found that low vitamin D levels are linked to hospitalization of SARS‐CoV‐2‐infected patients. Moreover, serum vitamin D levels lower than 16 ng/mL have been associated with an enhanced threat of sepsis among critically ill patients.^55^ The deficiency of vitamin D concentrations in serum concentrations quadrupled the likelihood of hospitalization owing to SARS‐CoV‐2 infection according to a large Israeli study cohort. A retrospective case study indicated that the serum concentrations of vitamin D among COVID‐19 patients were lower in critical cases compared with acute cases.^56^ Not only deficiency factors but also several key parameters are responsible for the severity of infections in the elderly population, including ethnicity, male sex, socioeconomic factors, and concurrent diseases such as obesity, diabetes mellitus, and hypertensive states.^57^ A pilot investigation (randomized clinical trial) stated in October 2020 that higher doses of Calcifediol (25(OH)D) can considerably decrease the viral load of ICU patients^58^ and a clinical case series concluded that those who took high doses of vitamin D showed enhanced clinical recovery, lower oxygen requirement, and fewer days in hospital.^52^ In addition, a study with realistic trial design analyzed in parallel the vitamin D3 states in the early stages of infection. The main motive of vitamin D (1,25(OH)_2_D3) supplementation is to boost the immune defense mechanism by decreasing pro‐inflammatory cytokine production.^59^ Approximately 24% of US citizens and 37% of Canadians were found to be suffering from vitamin D deficiency.^60^ Systematic reviews and meta‐analyses have illustrated that vitamin D can prevent respiratory infections. A retrospective multicenter study has reported that patients with vitamin D deficiency usually had worse results than patients with high vitamin D concentrations.^61^

## 6 REGULATION OF AUTOPHAGY BY VITAMIN D

Autophagy is an essential role of the immune system because it safeguards against viral infections.^62^ 1,25(OH)_2_D3 influences autophagy by modulating various approaches, including the generation of apoptogenic molecules such as Bcl‐2 along with cell regulatory mechanisms including the mammalian target of rapamycin pathway, class III phosphatidylinositol 3‐kinase complex (an enzyme involved in cellular growth proliferation and differentiation), and cathelicidin biosynthesis. As a result, it accelerates virus clearance and diminishes the viral burden. Vitamin D suppresses SARS‐CoV‐2 replication by increasing autophagy in macrophages.^63^ Resistance to SARS‐CoV‐2 may indeed be established with VDR and enough vitamin D.^29^

## 7 IMMUNOMODULATORY FUNCTION OF VITAMIN D: IMMIGRATION OF NEUTROPHILS

Patients infected with SARS‐CoV2 frequently display a change in neutrophil percentage. Neutrophils are specialized cells that cause phagocytosis to protect early responses towards invading pathogens.^64^ Microbicidal processes, phagocytic pathways, degranulation, and transfer of extracellular traps in neutrophils contribute to strengthening innate immune responses against infectious illnesses. Because neutrophil extracellular traps include specialized proteolytic enzymes that can degrade bacterial virulence factors, they have antibacterial activity. Neutrophil extracellular traps defend the host by increasing IL‐1 and interferon‐γ in activated neutrophils.^65^

## 8 VITAMIN D DEFICIENCY: EFFECTS ON OXIDATIVE AND NITRATIVE STRESS

The inhibition of oxidative stress provides a promising effect against antiviral infection. Moreover, excessive induction of the oxidative mediators is linked to pathophysiology and tissue damage mechanisms.^66^ The phosphatidyl inositol 3‐kinase signaling cascade decreases reactive oxygen species (ROS) and inducible nitric oxide synthase (iNOS) in immune cells, including monocytes and macrophages. The elegance III phosphatidylinositol 3‐kinase complex is an important signaling pathway regulated by 1,25(OH)_2_D3. As a result, 1,25(OH)_2_D3 plays a function in redox homeostasis in ROS and iNOS induction to improve antiviral activity and anti‐oxidative hindrance of iNOS, and induction of ROS scavenging pathways to block immunopathology.^32^

## 9 THERAPEUTIC EFFECTS OF VITAMIN D IN COVID‐19 PATIENTS

Age, genetic predispositions, and pre‐existing chronic diseases are also responsible for the respiratory diseases associated with COVID‐19.^13^ Vitamin D deficiency is also more common among the elderly and in communities receiving a low amount of vitamin D because of reduced vitamin D uptake and metabolism, mainly in African Americans.^67^ Similarly, inadequate vitamin D levels are also prevalent in prolonged incurable diseases such as cardiovascular disease and hyperglycemia, leading to a possible indirect connection between vitamin D inadequacy and high risk of COVID‐19.^68^ The previous study has also revealed that SARS‐CoV‐2‐infected patients living in South Asia and Switzerland have respiratory complications due to their vitamin D‐deprived state.^69^ Another association was observed between vitamin D and SARS‐CoV‐2, where enhanced mortality rates were observed in northern US states that received less UVB compared with southern regions. Similarly, global data have shown greater rates of mortality where only an average vitamin D supplement is taken.^70^ Vitamin D insufficiency is linked to several infections, including human immunodeficiency virus, tuberculosis, dengue, malaria, leprosy, as well as multiple sclerosis, inflammatory bowel disease, and cancer.^71^ Low levels of vitamin D are also linked to hepatitis C and liver fibrosis.^72^ Two randomized controlled trials (RCTs) report that proper supplementation of vitamin D can provide beneficial results against seasonal influenza.^73^ A few limitations were seen in the RCTs, which included various individuals vaccinated against influenza who were given vitamins but showed no beneficial effect.^74^ Levels of vitamin D above 50 ng/mL (125 nmoL/L) might be promising in decreasing the severity of COVID‐19.^75^ Malek et al describe the role of vitamin D in the modulation of the detrimental endocrine renin‐angiotensin system and downregulated renin synthesis. It seems to restrict renin and the ACE axis, thus improving the expression of ACE2, Mas receptor (MasR), and Ang (1‐7) concentration, that play an important role in the amelioration of acute lung injury. Therefore, vitamin D could be a promising advance in the treatment of COVID‐19 and ARDS.^76^ It can influence the activity of the ACE2/Ang (1‐7)/MasR and ACE2/Ang (1‐7) axes. It appears that vitamin D prophylaxis (avoiding overdosing) could help to lessen severity of COVID‐19, particularly in cases of vitamin D insufficiency.^77^ Tsujino et al demonstrated that in lung tissue, activation of vitamin D3 results in a protective action against interstitial lung disease.^78^ Martineau et al^47^ also showed that a regular dose of approximately 2000 IU/day with no additional bolus requirement showed safe and protective action against the virus.

### 9.1 Function and outcomes of vitamin D in SARS‐CoV‐2 infections in other studies

Several clinical trials and cohort studies indicate the beneficial contribution made by vitamin D in SARS‐CoV‐2 infection. However, a few retrospective observational studies suggest an inter‐relationship between vitamin D and the associated SARS‐CoV‐2 infection. For example, a retrospective study conducted on SARS‐CoV‐2 among 212 patients from three different hospitals in South Asia that evaluated the vitamin D levels in collected serum samples concluded that the level of vitamin D was significantly different in four conditions; mild (78 nmol/L), ordinary cases (68.5 nmol/L), severe cases (53 nmol/L), and critically affected COVID‐19 patients, where the level was too low. The authors of this study indicated the significant correlation among clinical outcomes (*P* < 0.001) underlying vitamin D status. In Singapore, a small cohort study was conducted^79^ among 43 SARS‐CoV‐2‐positive patients who were given a combination therapy of oral vitamin D dose of 1000 IU, magnesium (150 mg), and vitamin B12 (500 μg) and essential oxygen compared with a control group (3 out of 17 vs 16 out of 26; *P* = 0.006). The findings concluded that the combination treatment of vitamin D, magnesium, and vitamin B12 exerted a considerable defense against clinical pathophysiology due to infection (*P* = 0.041). Decrease in vitamin D concentration was also observed in critically ill COVID‐19 patients with a pre‐existing medical history.^80^ In one of the retrospective observational studies conducted among 186 SARS‐CoV‐2‐positive survivors of COVID‐19, vitamin D levels were found to be significantly lower (*P* = 0.0016) when compared with the control group. Another study collected data sets from various regions of the world and reported a 15% decrease in disease rate after taking vitamin D supplementation.^81^ In Indonesia, a retrospective cohort study was performed among 780 patients and found a higher death rate in patients with low levels of vitamin D.^82^ Another retrospective study from the USA concluded that sunlight and vitamin D might reduce the risks of SARS‐CoV‐2 infection.^83^

## 10 BENEFICIAL EFFECTS OF VITAMIN D UPTAKE

According to previous evidence, no data indicates that supplementing vitamin D can minimize the relative severity and high mortality rate of COVID‐19. Very few randomized clinical trials have been registered on the SARS‐CoV‐2‐infected population that compare their vitamin D levels. A small cohort study was conducted that validated the promising results of vitamin D in combination with vitamin B12 and magnesium among the SARS‐CoV‐2 infected patients. Regarding respiratory tract infection prevention, a meta‐analysis demonstrated that dietary vitamin D administration is a safe and robust agent against SARS‐CoV‐2 infections.^84^ In addition, the study concluded that those with lower levels of vitamin D took the most advantage from this supplementation. In another identical study, subgroup evaluation specified that each daily or weekly consumption of dietary vitamin D (not more than extra bolus doses) could protect from acute upper respiratory infection, specifically in patients with a lack of dietary vitamin D. Vitamin D administration enhanced gene expression associated with anti‐oxidation (glutathione reductase modifier subunit).^85^ Indeed, treatment of adults with vitamin D (100 μg/day) is safe.^36^, ^86^ The heterogeneity of the population and the dose regimens of vitamin D must be considered in the prevention of SARS‐CoV‐2 infections. Checking the influence of nutrition D administration in RCTs, and a serious serum‐based study of 25(OH)D instead of administered vitamin D dose are suggested.^87^

## 11 VITAMIN D AND COVID‐19: SUMMARY OF THE EVIDENCE

### 11.1 Overview

**FIGURE 4:**
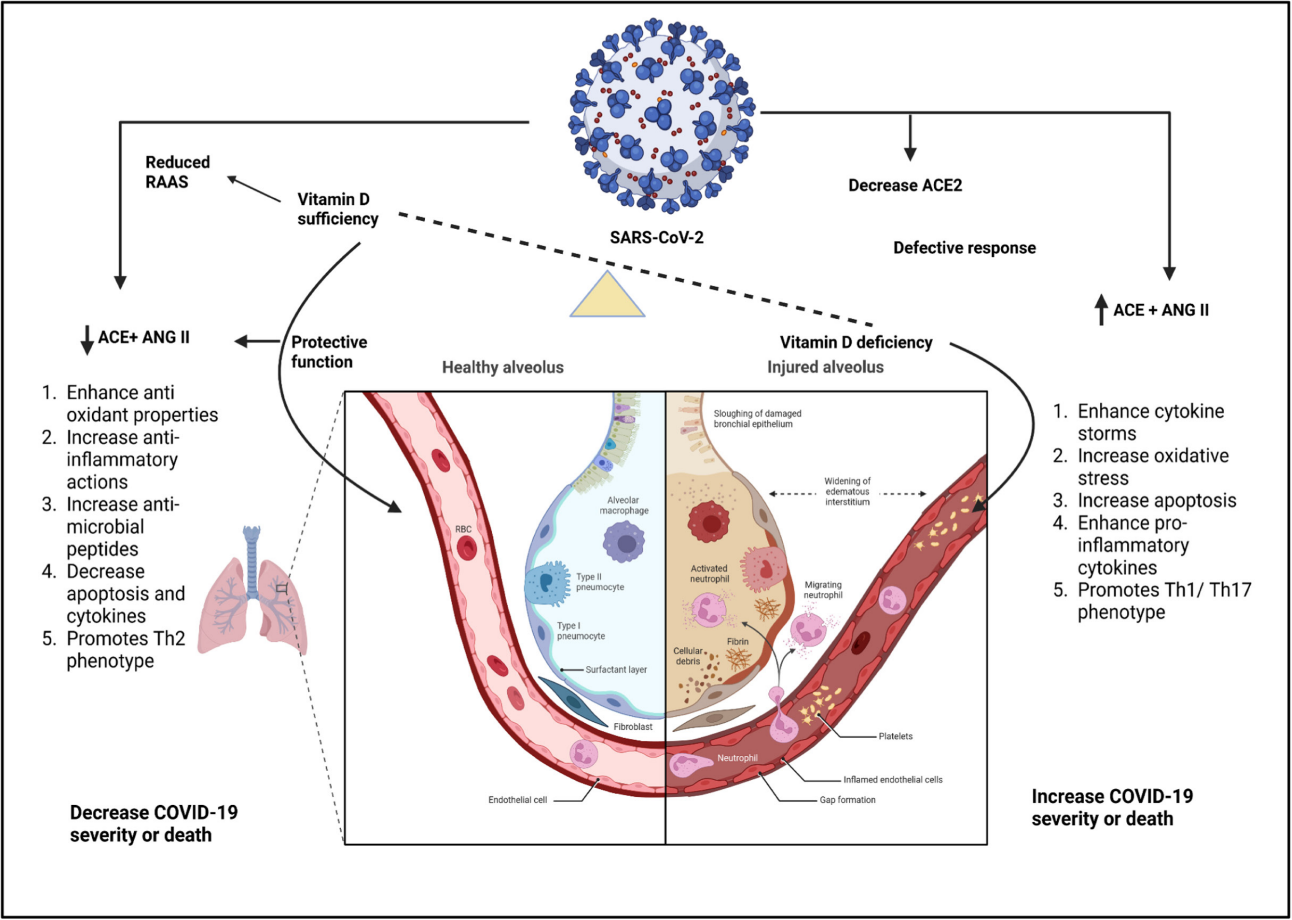
Possible methods by which adequate serum vitamin D levels could protect against COVID‐19 and acute lung injury, whereas vitamin D deficiency might cause a faulty immunological response to COVID‐19, leading to increased severity and/or fatality. Arrows pointing up or down denote a rise or reduction, respectively

The alleged function of vitamin D in the management or therapy of COVID‐19 is dynamic and complex. As previously mentioned, vitamin D has the ability to modulate various components of immunity, potentially affecting the severity and consequences of COVID‐19. Downregulation of ACE2 brought on by SARS‐CoV‐2 infections results in toxic Ang II build‐up, which in turn causes ARDS. It has been discovered that vitamin D reduces the consequences of these interactions between SARS‐CoV‐2 and the renin‐angiotensin‐aldosterone system (RAAS).^88^ Vitamin D can activate the vasorelaxant ACE2/Ang (1‐7)/Mas receptor axis, a negative endocrine regulator on the RAAS that guards against acute lung injury and ARDS (Figure 4). By attaching to the VDR and suppressing the expression of renin‐producing enzymes and proteins, vitamin D inhibits the manufacture of renin.^89^ It has also been demonstrated that vitamin D promotes ACE2 expression. Data suggest that differing expressions of ACE2, which generate a heightened and more robust immunological response in females, may be the reason for the differential presentation of COVID‐19 between male and female patients (higher likelihood of ICU admissions and death in males).^90^ Because males express ACE2 at higher levels than females, its impact on the severity of COVID‐19 has been assessed.^91^ A study employing an animal model discovered that females following oophorectomy showed higher ACE2 activity compared with before, demonstrating that many of these alterations can be linked to the presence of estrogen.^92^ In essence, COVID‐19 is more severe in men because they express ACE2 more and do not benefit from estrogen's cardioprotective effects. Although intricate mechanisms for the cardiovascular consequences of COVID‐19 have been put forward,^93^ it is apparent that damage has been done.^94^ ACE2 and the RAAS have been linked in the process by which COVID‐19 substantially increases the strain on the cardiovascular system, and individuals who already have cardiovascular morbidities are more susceptible to problems and mortality. In a case study of 43 patients in China, it was discovered that men were more likely than women to have more severe COVID‐19.^95^ This was also found in the USA and Europe.^96^ Different amounts of 1,25(OH)_2_D3 and VDR expression may have a role in the disparities between males and females, according to other observational research.^97^ Beyond the mechanistic explanation of how 1,25(OH)_2_D3 signaling modifies the immune response, women also benefit from vitamin D's synergistic action with estrogen in CD4^+^ T cells, which inhibits an autoimmune response.^98^ However, it might be an oversimplification to attribute gender variations in COVID‐19 to ACE2 or vitamin D. This could be explained by fewer morbidities, in a manner that may be very varied because of individual variations, as estrogen confers a protective effect on cardiovascular and immune health.^99^ It is likely that low vitamin D levels cause cytokine storms, poor defense against epithelial cell death, and inadequate epithelial cell repair, ultimately leaving the lungs susceptible to catastrophic immune system dysregulation. It is expected that vitamin D is a more effective immune system modulator than antibody treatment for halting the function of cytokines like IL‐6.^100^ The action of the TMPRSS2 is likely to be inhibited by vitamin D and its physiologically active hydroxy derivatives.^101^ As a result, the fusion of the viral spike protein and ACE2, which is necessary for viral entry into the host cell, is blocked. The physiologically active compounds that stop the serine proteases from interacting with each other are involved in a variety of biological processes. The involvement of vitamin D in COVID‐19 has also been verified by research employing genomics guided tracing to identify the targets of SARS‐CoV‐2. Glinsky^102^ investigated vitamin D as a putative inhibitor of ACE2 expression and discovered that vitamin D appears to decrease ACE2 expression by way of the VDR and other transcription factors in human bronchial smooth muscle cells. Vitamin D influences expression levels of 84 of the 332 SARS‐CoV‐2 prey protein‐coding genes (25%). These prey proteins perform a variety of cellular processes that are interfered with by infection. This shows that vitamin D has the ability to interfere with the function of 19 out of 27 (or 70%) of the SARS‐CoV‐2 proteins, in addition to blocking the production of ACE2. Mechanistic investigations using transcriptome or metabolomic analysis are eagerly awaited to validate the involvement of vitamin D in SARS‐CoV‐2 infections. However, numerous observational studies and open‐label, RCTs have demonstrated a substantial relationship between vitamin D and COVID‐19 **(**Table 1).^52^, ^59^, ^62^, ^67^, ^103^, ^104^, ^105^, ^106^, ^107^, ^108^, ^109^, ^110^, ^111^, ^112^, ^113^, ^114^, ^115^, ^116^, ^117^, ^118^, ^119^, ^120^, ^121^, ^122^, ^123^, ^124^, ^125^, ^126^, ^127^, ^128^, ^129^, ^130^, ^131^, ^132^, ^133^, ^134^, ^135^, ^136^, ^137^, ^138^, ^139^, ^140^, ^141^, ^142^, ^143^

**TABLE 1:** Characteristics of studies examining the links between vitamin D and COVID‐19

Study design	Sample size	Main findings	References
Correlation analysis of data	88 countries	Countries lying near to the equator had fewer COVID‐19 fatalities than those lying farther away; latitude accounted for 16% of this difference	103
Cohort study	105	Lack of vitamin D was linked to increased care requirements and the development of cytokine storms	104
Retrospective cohort study	107	SARS‐CoV‐2‐positive individuals showed lower levels of 25(OH)D	105
Retrospective cohort study	4314	A higher risk of COVID‐19 was associated with inadequate vitamin D levels	106
Retrospective study	185	Lack of vitamin D was linked to an increased risk of invasive mechanical ventilation or death	107
Randomized prospective open‐label study	87	In people with hypovitaminosis D, vitamin D supplementation reduced inflammatory markers	108
Retrospective cohort study	56	A lack of vitamin D in patients with COVID‐19 reported greater levels of inflammatory markers along with considerably reduced level of hemoglobin and lymphocyte counts	52
Parallel pilot randomized open‐label trial	76	In patients who needed to be admitted to the ICU, high dosage calcifediol lessened the severity of COVID‐19	109
Multi‐center parallel double‐blind RCT	240	When compared with placebo, a single high dose of cholecalciferol had no effect on hospital stay, death, ICU admission, or the need for ventilation	110
RCT	40	A higher percentage of asymptomatic or slightly symptomatic patients on high dosage vitamin D—25(OH)D >50 ng/mL achieved a negative SARS‐CoV‐2 RNA at less than 21 days—than those with vitamin D deficiency	111
Quasi‐randomized trial	66	In an elderly population, vitamin D administration before or after COVID‐19 decreased disease severity and fatality rates	112
Systematic review and meta‐analysis	6 retrospective articles	A helpful prognostic indicator of COVID‐19 results could be vitamin D levels	113
Living Cochrane systematic review	3 RCTs	The available information is insufficient to definitively determine the advantages or disadvantages of vitamin D supplementation as a COVID‐19 treatment	114
Open‐label, multicenter, superiority RCT	NA	In SARS‐CoV‐2‐positive patients exhibiting at least one symptom of elevated risk, a single dose of 50 000 IU of vitamin D was compared with a single dose of 200 000 IU of vitamin D. Incomplete results	115
Quasi‐experimental study	77	Before COVID‐19, regular vitamin D administration decreased mortality in elderly individuals at the 3‐month follow up	116
Systematic review and meta‐analysis	21 studies	Low vitamin D levels are correlated with severe COVID‐19 illness, which may be explained by the fact that 25(OH)D is inversely correlated with pro‐inflammatory cytokines like interleukin‐6, an increase in C‐reactive protein, and cardiac insufficiency, which are related to the severity of COVID‐19 and its unfavorable outcomes. A 25(OH)D shortage and sensitivity to infection by COVID‐19 have not been linked causally, despite a linkage between higher vitamin D levels, immunological responses, and a better prognosis in other viral infections, according to testing and blood vitamin D tests in SARS‐CoV‐2 patients	62, 117, 118
Cohort study	43	If they did not need oxygen therapy, patients were given 500 μg/day of vitamin B12, 150 mg/day of magnesium, and 1000 IU/day of vitamin D3 (DMB) when they were admitted. Patients who received DMB had much less deterioration to the point of needing oxygen therapy or intensive care support, even after adjusting for demographics and hypertension	119
Retrospective cohort study	499	The incidence of COVID‐19 was associated with vitamin D deficiency status in addition to age and non‐white race. The likelihood of testing positive for COVID‐19 was not substantially associated with the amount of vitamin D supplementation	120
Retrospective observational analysis	191 779	Patients with inadequate 25(OH)D scores had a greater incidence of SARS‐CoV‐2 than patients with sufficient values and those with levels under 55 ng/mL	121
Observational study	154	Patients who were asymptomatic had mean vitamin D levels that were noticeably higher than patients who were gravely unwell. In people who were extremely unwell, vitamin D deficiency was more common. 90 out of 154 patients had vitamin D deficiency (29 asymptomatic; 61 severely ill). Patients with vitamin D deficiency had greater levels of inflammatory markers, an inflammatory response, and a higher mortality rate in their serum (21% vs 3.1%). Patients with severe COVID‐19 had noticeably low levels of vitamin D	122
Retrospective observational study	42	Patients with severe vitamin D deficiency had a 50% chance of dying after 10 days in the hospital, whereas those with insufficiency or moderate deficiency had a 5% chance. Hypovitaminosis D was found to be highly prevalent in 19 patients with acute respiratory failure who were receiving treatment in the ICU, which was associated with a significant mortality risk	123
Observational study	NA	Government statistics data on mortality were inversely associated with the mean vitamin D levels reported in various states and territories	124
Retrospective observational study	444	A lower incidence of COVID‐19 mortality was associated with the administration of cholecalciferol booster	125
Retrospective observational trial	186	Independent of age, chronic lung illness, and the severity score from the chest computed tomography, vitamin D deficiency at admission was linked to mortality	126
Retrospective observational study	149	A lower level of serum 25(OH)D was linked to higher mortality in COVID‐19 patients	127
Retrospective observational study	464	After sex was ruled out as a factor in COVID‐19 severity or fatality, levels of 25(OH)D <12 ng/mL were found to be strongly associated with an elevated risk of fatal disease and severe sickness	128
Cohort study	646	There is no proven link between low vitamin D levels and COVID‐19 severity or fatality	129
Observational cohort study	445	There was a link between hypocalcemia and the severity of COVID‐19, but there was no linkage between vitamin D and the severity or advancement of the disease. It is also accepted that calcium and vitamin D interact	130
Retrospective observational study	1549	But not mortality, hospital admission and critical care were associated with low vitamin D levels	131
Retrospective cohort study	144	The level of serum vitamin D was negatively linked with hospital mortality and the requirement for mechanical ventilation	132
Prospective observational study	410	There is no link between a vitamin D deficiency and the prevalence of severe COVID‐19, a rise in oxygen use, hospital admissions, or death. There was no appreciable change in the outcome in vitamin D‐deficient patients treated with cholecalciferol	133
Retrospective, multicenter, non‐randomized cohort study	537	After COVID‐19 diagnosis, calcifediol medication was significantly linked to a lower 30‐day mortality rate	134
Observational study	157	It was discovered that vitamin D supplementation had an adverse relationship with mortality in COVID‐19 patients	135
Cohort study	1486	The incidence of COVID‐19 was higher in patients with Parkinson's disease who were younger, heavier, and had chronic obstructive pulmonary disease. Patients who took vitamin D supplements had a lower likelihood of developing it	136
Retrospective study	91	In patients with comorbidities, two doses of 200 000 IU of vitamin D given on consecutive days can enhance outcomes (ICU admission, mortality)	137
Case‐control study	60 039	Statins that raised levels of 25(OH)D, such as rosuvastatin, were the only ones to have a protective effect in COVID‐19	138
Retrospective survival study	16 401	Following the administration of vitamin D (calcifediol) 15‐30 days before hospitalization, there was a significant decrease in mortality	139
RCT	69	For 2 weeks, patients with mild to severe symptoms of COVID‐19 required less time to recover when given 5000 IU of vitamin D daily rather than 1000 IU. Over time, the interleukin‐6 and body mass index levels in both groups decreased	140
Population‐based cohort study	108 343	Infection rates were more likely to increase with lower serum 25(OH)D levels than disease severity or mortality. Patients with COVID‐19 who received vitamin D supplements compared with those who did not receive any supplements	59
Clinical case series	4	Treatment with calcifediol significantly decreased mortality and ICU admission in hospitalized COVID‐19 patients	141
Observational study	838	Treatment with calcifediol significantly decreased mortality and ICU admission in COVID‐19 patients who were hospitalized	142
Prospective study	8297	A decrease in COVID‐19 incidence was associated with routine vitamin D intake	67

Abbreviations: COVID‐19, coronavirus disease 2019; RCT, randomized controlled trial; SARS‐CoV‐2, severe acute respiratory syndrome coronavirus 2.

### 11.2 Observational studies

There have been various observational studies looking into the role that vitamin D plays in SARS‐CoV‐2 infections. A significant cross‐sectional study that looked at the geographic distribution of COVID‐19 in the USA discovered associations between outcomes including illness severity and COVID‐19 deaths and sunlight exposure as a proxy for vitamin D.^103^ In a cohort study of an elderly population, it was found that patients with COVID‐19 who were admitted to a hospital in the UK had lower serum levels of vitamin D compared with healthy controls, and that there was a pronounced correlation between vitamin D deficiency and needing more care or ventilation. Additionally, Baktash et al,^104^ reported that a lack of vitamin D is linked to a higher frequency of cytokine storms. Similarly, those who tested positive for SARS‐CoV‐2 were more likely to have lower circulating 25(OH)D concentrations in two trials involving 107 and 4314 patients in Switzerland and Chicago, respectively.^107^ Others have discovered links between having enough vitamin D and a lower incidence of COVID‐19, but not in terms of what happens following infection. Some research, however, has presented contradictory, unfavorable findings. Overall, the data point to the significance of the association between vitamin D and COVID‐19, an association that calls for further research through large‐scale, nationally representative investigations. There are obviously many confounding variables in observational data, and up to this point, there have not been any randomized controlled studies to determine whether vitamin D specifically has a role in COVID‐19 susceptibility and consequences. Notably, this narrative review presents some recent associative evidence on the relationship between vitamin D and COVID‐19. One questionnaire‐based study of 1486 Parkinson's patients from Italy found that those who used vitamin D supplements had a lower risk of having COVID‐19.^144^ Patients who tested positive for SARS‐CoV‐2 by polymerase chain reaction had decreased serum levels of vitamin D, according to a second small Italian investigation (*n* = 107).^143^ The chance of testing positive for COVID‐19 rose when vitamin D levels were low, according to a third study from the USA (*n* = 4314).^119^

### 11.3 Clinical trials

In addition to receiving normal therapy, supplementing with vitamin D significantly reduced the inflammatory markers in 87 patients with COVID‐19 and hypovitaminosis D, according to a randomized prospective open‐label study conducted in India.^108^ In comparison to individuals receiving no supplements, levels of C‐reactive protein, lactate dehydrogenase, IL‐6, ferritin, and neutrophil to lymphocyte ratios significantly decreased in the patients receiving 60 000 IU of vitamin D supplementation daily for 8 days. A retrospective cohort research that included COVID‐19 patients who were vitamin D deficient found that they had considerably lower lymphocyte and hemoglobin counts and higher levels of inflammatory markers, such as C‐reactive protein.^52^ In addition, patients with vitamin D deficiencies were more likely to need oxygen therapy and were more likely to develop pneumonia if their vitamin D levels had not been raised 6 months before being infected by SARS‐CoV‐2. The effectiveness of oral calcifediol, azithromycin, and hydroxychloroquine in treating COVID‐19 was examined in an RCT in Spain. They reported that adequate vitamin D levels brought on by calcifediol supplementation were the most obvious determinant in determining disease prognosis, even when hydroxychloroquine and azithromycin were given in accordance with standard of care treatment. In an RCT conducted in India by Rastogi et al,^110^ it was discovered that asymptomatic or mildly symptomatic patients could achieve a negative SARS‐CoV‐2 RNA by day 21 when given high‐dose vitamin D (60 000 IU of daily cholecalciferol for 7 days), achieving a therapeutic target of 25(OH)D greater than 50 ng/mL. In contrast, a different RCT in Brazil found that among 240 hospitalized COVID‐19 patients, a single high dose of 200 000 IU of cholecalciferol had no difference from placebo in terms of reducing hospital length of stay, in‐hospital mortality, admission to the intensive care unit, or need for mechanical ventilation. It should be emphasized that individuals in this trial were given a variety of concurrent drugs and vitamin D after a considerable amount of time had passed since the onset of symptoms (mean 10.3 days). Therefore, it is uncertain if the delayed results may be to blame and whether early or preventive vitamin D treatment may be helpful in treating mild or moderate COVID‐19. In fact, it was found that vitamin D administration during or immediately before COVID‐19 was related to less severe disease and reduced mortality rates in 66 elderly residents of a French nursing home participating in quasi‐randomized research looking into possible infection prevention.^111^ The National Institute for Health and Care Excellence assessment includes 12 observational studies examining relationships between serum vitamin D concentrations and the occurrence or treatment of COVID‐19, one small RCT of vitamin D as a therapy, and no trials of vitamin D as a preventive measure.^145^

## 12 RECOMMENDED DOSE

Several recommended doses have been approved depending on various circumstances. Table 2 describes the probable doses.^13^, ^146^, ^147^, ^148^, ^149^, ^150^, ^151^

**TABLE 2:** Recommended vitamin D dose

S. No	Suggested doses
1.	With the slight increase in serum concentration of 25(OH)D, the degree of protection also increases; however, the optimal range lies between 40‐60 ng/mL (100‐150 nmol/L). To gain these levels, approximately 2000‐5000 IU/day of vitamin D intake is required in 50% of the population^145^
2.	To sustain 30 ng/mL of the serum concentration of 25(OH)D, administration of several loading doses is needed. One study employed a dose of 100 000‐200 000 IU weekly for 8 consecutive weeks (1800 or 3600 IU/day)^146^
3.	Clinical evidence suggests that either weekly or daily consumption of vitamin D is more efficacious than bolus doses in patients experiencing lung infections. Still, a substantial intake of vitamin D can indeed be deleterious and toxic^147^, ^148^
4.	To avoid infection, some studies suggest taking a high amount of vitamin D once a day^149^
5.	High doses (600 000 IU administered orally once) should therefore be avoided because they significantly increase the chance of toxicity
6.	According to the previous research, taking 10 000 IU every day for 1 month will enhance serum 25(OH)D concentrations into the ideal range of 40‐60 ng/mL.^150^ To maintain that level, the dose is lowered to 2000‐3000 IU/day after the first month
7.	Patients hospitalized with COVID‐19 should have initial basal serum 25(OH)D amounts evaluated and supplemented to a level of approximately 30 ng/mL (ideally 40‐60 ng/mL), specifically if the baseline level is far less than 10 ng/mL, as this deficit is substantially more prevalent in male patients^151^
8.	In SARS‐CoV‐2‐infected patients with 25(OH)D serum concentrations <20 ng/mL, the suggested dose is 6000‐7000 oral IU/day for 6‐8 weeks. For protection, the dose may vary from 2000‐3000 oral IU/day depending on the age and clinical situation
9.	Vitamin D administration is required when the baseline plasma concentrations of SARS‐CoV‐2‐infected hospitalized patients are <30 ng/mL (optimal 40‐60 ng/mL), especially when the baseline level is 10 ng/mL
10.	While baseline 25(OH)D concentrations in infected patients cannot be determined, 2000‐3000 oral IU per day is suggested

## 13 LIMITATIONS AND FUTURE DIRECTIONS

The rapid evolution of SARS‐CoV‐2 and its newly appearing, little known impacts on the immune system severely restrict the field's ability to advance. Re‐examining what little is known is necessary in the light of this phenomenon where more infectious or virulent virus strains result in negative effects even in younger populations that are considered to be more immune‐resilient. Even though the evidence now available strongly supports the concept that vitamin D plays a role, it is not conclusive; and some studies have found no connection between vitamin D and COVID‐19 when other confounding variables are taken into account.^152^ Furthermore, the underlying etiology of the vitamin D deficiency, and consequently, the immunological dysfunction identified in COVID‐19, are still up for debate. It has also been suggested that, rather than a shortage of vitamin D itself, diabetes and obesity (both of which are accompanied by vitamin D‐deficient states) are to blame for the rise in COVID‐19 mortality.^153^ Others make the case for the “healthy user effect,” which states that people with healthy diets and regular exercise spend more time outside and have better vitamin D levels as a result. This demonstrates associations, and not causalities, between vitamin D insufficiency and poor COVID‐19 outcomes. Taken together, these differences make it impossible to draw firm conclusions and emphasize the necessity of well thought out, appropriately powered experiments to ascertain the function of vitamin D in COVID‐19. There are currently 21 active trials that could provide some insight into this subject in the near future, according to a recent Cochrane review. Vitamin D deficiency has also been advised to be treated preventatively as it lessens the severity of disease following infection. This needs to be taken into account and needs further examination in light of other risk factors like aging and age‐related health degradation.^19^ Overall, there is still much left to be done to assess the immunological implications of the mutant SARS‐CoV‐2 strains. Additionally, more research has to be done on the mechanistic processes underpinning the cellular and molecular activities of vitamin D concentrations in the context of COVID‐19.

## 14 SIGNIFICANCE OF THE VITAMIN D AND SARS‐COV‐2 STUDIES

There has been plenty of discussion on the effect of vitamin D on SARS‐CoV‐2 infection, hospitalization, and mortality rates. Vitamin D deficiency is likely to be a significant factor in transmission and complications, according to a large body of evidence, including known COVID‐19 immunity pathways, vitamin D physiology and its effects on the immune system, and population‐based studies linking vitamin D levels to respiratory infections.^154^ Observational studies comparing outcomes across nations show an inverse relationship between vitamin D status and the severity of COVID‐19 disease and its associated mortality, suggesting that vitamin D may influence the immune system's response to infection.^155^ In particular, Spain and Italy have recorded the highest rates of COVID‐19 infection and deaths worldwide, as well as high rates of vitamin D insufficiency. In contrast, the Nordic nations have a lower incidence of COVID‐19 infection and death as well as higher levels of vitamin D due to systematic dietary fortification. Other statistics, however, cast doubt on such a connection. For instance, Brazil, a country on the equator, has high rates of both cases and mortality from COVID‐19, whereas Greece, a country with a prevalence of vitamin D deficiency (25(OH)D 20 ng/mL) of at least 50% over a wide age range,^156^ is among the nations with the lowest numbers of confirmed COVID‐19 cases and deaths. The results of well‐conducted RCTs demonstrating the impact of vitamin D on COVID‐19 clinical outcomes should be awaited by the entire world. It is only reasonable to wonder if vitamin D could prevent COVID‐19 given that the earlier research studies have shown that it could prevent acute respiratory infection. This topic was covered by Rhodes et al in a narrative review that was published in the *Journal of Internal Medicine*.^157^ The authors compared the mortality of COVID‐19 in relation to the latitude of various nations in order to first establish a definitive association between vitamin D levels and COVID‐19. After adjusting for age, they discovered a 4.4% rise in mortality for every degree of latitude north of 28°. This finding suggests that indirect vitamin D from UV light may have a role in COVID‐19 protection. Furthermore, they draw attention to the fact that risk variables for vitamin D insufficiency overlap with those linked to COVID‐19 death (old age, ethnicity, male sex, obesity, diabetes, and hypertension). The “healthy user effect,” which states that healthier people just spend more time outside and eat more than people who are less healthy, may entirely explain this overlap, despite the fact that it is striking and merits further study. Although there is no direct evidence linking vitamin D levels to the occurrence or results of COVID‐19, there is indirect evidence that vitamin D has an immunomodulatory role in respiratory infections. The similarity of the risk variables for severe COVID‐19 and vitamin D deficiency, including older age, obesity, and minority ethnicity, constitutes additional indirect evidence. In high‐latitude nations, there is a link between the seasonal drop in vitamin D serum concentrations and a larger burden of COVID‐19.^158^ Together, the available data make a strong justification for additional studies.

## 15 CONCLUSION

It has been observed that supplementation of vitamin D plays a major role in the prevention of infections. The defensive role of vitamin D against infections related to the respiratory system has been proven in various randomized clinical trials and meta‐analyses. It has been suggested that vitamin D supplementation be administered in the current pandemic situation to sustain the optimal levels of 25(OH)D circulating in the body (75‐125 nmol/L). Several studies indicate the association of COVID‐19 cases with abnormal vitamin D levels. Maintaining vitamin D serum concentrations (40 and 60 ng/mL) over the year may decrease the risk of respiratory tract infections and SARS‐CoV‐2 infections. Depending on the pandemic wave, large‐scale clinical trials may provide a more accurate picture of the relation between vitamin D deficiency and SARS‐CoV‐2. More studies are needed to study the role of vitamin D on the replication of SARS‐CoV‐2 and the inflection of ACE2. Immune responses against the virus can be achieved by regulating the vitamin D‐dependent mucosal microbiota, particularly in cystic fibrosis cases. Vitamin D is proven to possess multiple immune‐modulating actions, so it seems crucial to consider the prophylactic supplementation and co‐administration of vitamin D to invoke an effective therapeutic benefit in the modulation of the immune system and reduce the severity of SARS‐CoV‐2 infection, especially in elderly patients. Yet, more clinical research, including randomized and controlled clinical trials and large‐scale cohort investigations, are essential to further understand the therapeutic mechanisms of vitamin D in COVID‐19.

## CONFLICT OF INTEREST

The authors declare that there are no conflicts of interest.

## References

[1] Magrone T , Magrone M , Jirillo E . Focus on receptors for coronaviruses with special reference to angiotensin‐converting enzyme 2 as a potential drug target‐a perspective. Endocr Metab Immune Disord. 2020;20(6):807‐811. 10.2174/1871530320666200427112902 32338224

[2] Zhu N , Zhang D , Wang W , et al. A novel coronavirus from patients with pneumonia in China, 2019. N Engl J Med. 2020;382(8):727‐733. 31978945 10.1056/NEJMoa2001017 PMC7092803

[3] Di Rosa M , Malaguarnera M , Nicoletti F , Malaguarnera L . Vitamin D3: a helpful immuno‐modulator. Immunology. 2011;134(2):123‐139. 21896008 10.1111/j.1365-2567.2011.03482.x PMC3194221

[4] Manion M , Hullsiek KH , Wilson EM , et al. Vitamin D deficiency is associated with IL‐6 levels and monocyte activation in HIV‐infected persons. PLoS One. 2017;12(5):e0175517. 28464004 10.1371/journal.pone.0175517 PMC5413041

[5] Jose RJ , Manuel A . COVID‐19 cytokine storm: the interplay between inflammation and coagulation. Lancet Respir Med. 2020;8(6):e46‐e47. 32353251 10.1016/S2213-2600(20)30216-2 PMC7185942

[6] Holick MF . The vitamin D deficiency pandemic: approaches for diagnosis, treatment, and prevention. Rev Endocrine Metabolic Dis. 2017;18(2):153‐165. 10.1007/s11154-017-9424-1 28516265

[7] Dankers W , Colin EM , van Hamburg JP , Lubberts E . Vitamin D in autoimmunity: molecular mechanisms and therapeutic potential. Front Immunol. 2017;7:697. 28163705 10.3389/fimmu.2016.00697 PMC5247472

[8] Infante M , Ricordi C , Sanchez J , et al. Influence of vitamin D on islet autoimmunity and beta‐cell function in type 1 diabetes. Nutrients. 2019;11(9):2185. 31514368 10.3390/nu11092185 PMC6769474

[9] Bouillon R , Marcocci C , Carmeliet G , et al. Skeletal and extraskeletal actions of vitamin D: current evidence and outstanding questions. Endocr Rev. 2019;40(4):1109‐1151. 30321335 10.1210/er.2018-00126 PMC6626501

[10] Greiller CL , Martineau AR . Modulation of the immune response to respiratory viruses by vitamin D. Nutrients. 2015;7(6):4240‐4270. 26035247 10.3390/nu7064240 PMC4488782

[11] Gombart AF , Borregaard N , Koeffler HP . Human cathelicidin antimicrobial peptide (CAMP) gene is a direct target of the vitamin D receptor and is strongly up‐regulated in myeloid cells by 1, 25‐dihydroxyvitamin D3. FASEB J. 2005;19(9):1067‐1077. 15985530 10.1096/fj.04-3284com

[12] Wang TT , Dabbas B , Laperriere D , et al. Direct and indirect induction by 1, 25‐dihydroxyvitamin D3 of the NOD2/CARD15‐defensin β2 innate immune pathway defective in Crohn disease. J Biol Chem. 2010;285(4):2227‐2231. 19948723 10.1074/jbc.C109.071225 PMC2807280

[13] Grant WB , Lahore H , McDonnell SL , et al. Evidence that vitamin D supplementation could reduce risk of influenza and COVID‐19 infections and deaths. Nutrients. 2020;12(4):988. 32492787 10.3390/nu12061620 PMC7352449

[14] Watkins J . Preventing a covid‐19 pandemic. BMJ. 2020;368:m810. 32111649 10.1136/bmj.m810

[15] Daneshkhah A , Agrawal V , Eshein A , Subramanian H , Roy HK , Backman V . The possible role of vitamin D in suppressing cytokine storm and associated mortality in COVID‐19 patients. Med Rxiv. 2020.

[16] Darling AL , Ahmadi KR , Ward KA , et al. Vitamin D status, body mass index, ethnicity and COVID‐19: initial analysis of the first‐reported UK biobank COVID‐19 positive cases (n 580) compared with negative controls (n 723). Med Rxiv. 2020.

[17] De Smet D , De Smet K , Herroelen P , Gryspeerdt S , Martens GA . Vitamin D deficiency as risk factor for severe COVID‐19: a convergence of two pandemics. Med Rxiv. 2020.

[18] Hasti CE , Mackay DF , Ho F , et al. Vitamin D concentrations and COVID‐19 infection in UK biobank. Diabetes Metab Syndr. 2020;14(4):561‐565. 32413819 10.1016/j.dsx.2020.04.050 PMC7204679

[19] Ilie PC , Stefanescu S , Smith L . The role of vitamin D in the prevention of coronavirus disease 2019 infection and mortality. Aging Clin Exp Res. 2020;32(7):1195‐1198. 32377965 10.1007/s40520-020-01570-8 PMC7202265

[20] Lau FH , Majumder R , Torabi R , et al. Vitamin D insufficiency is prevalent in severe COVID‐19. Med Rxiv. 2020.

[21] Zemb P , Bergman P , Camargo CA Jr , et al. Vitamin D deficiency and the COVID‐19 pandemic. J Glob Antimicrob Resist. 2020;22:133–134. 32474141 10.1016/j.jgar.2020.05.006 PMC7256612

[22] Rondanelli M , Miccono A , Lamburghini S , et al. Self‐care for common colds: the pivotal role of vitamin D, vitamin C, zinc, and echinacea in three main immune interactive clusters (physical barriers, innate and adaptive immunity) involved during an episode of common colds—practical advice on dosages and on the time to take these nutrients/botanicals in order to prevent or treat common colds. Evid Based Complement Alternat Med. 2018;2018:5813095. 29853961 10.1155/2018/5813095 PMC5949172

[23] Bikle DD . Vitamin D metabolism, mechanism of action, and clinical applications. Chem Biol. 2014;21(3):319‐329. 24529992 10.1016/j.chembiol.2013.12.016 PMC3968073

[24] Schwalfenberg GK . A review of the critical role of vitamin D in the functioning of the immune system and the clinical implications of vitamin D deficiency. Mol Nutr Food Res. 2011;55(1):96‐108. 20824663 10.1002/mnfr.201000174

[25] Murdaca G , Pioggia G , Negrini S . Vitamin D and Covid‐19: an update on evidence and potential therapeutic implications. Clinic Mol Allergy. 2020;18(1):1‐8. 10.1186/s12948-020-00139-0 PMC7675394 33292313

[26] Adams JS , Ren S , Liu PT , et al. Vitamin d‐directed rheostatic regulation of monocyte antibacterial responses. J Immunol. 2009;182(7):4289‐4295. 19299728 10.4049/jimmunol.0803736 PMC2683618

[27] Laaksi I . Vitamin D and respiratory infection in adults. Proc Nutr Soc. 2012;71(1):90‐97. 22115013 10.1017/S0029665111003351

[28] Herr C , Shaykhiev R , Bals R . The role of cathelicidin and defensins in pulmonary inflammatory diseases. Exp Opinion Biol Therapy. 2007;7(9):1449‐1461. 10.1517/14712598.7.9.1449 17727333

[29] Yuk JM , Shin DM , Lee HM , et al. Vitamin D3 induces autophagy in human monocytes/macrophages via cathelicidin. Cell Host Microbe. 2009;6(3):231‐243. 19748465 10.1016/j.chom.2009.08.004

[30] Liu PT , Stenger S , Li H , et al. Toll‐like receptor triggering of a vitamin D‐mediated human antimicrobial response. Science. 2006;311(5768):1770‐1773. 16497887 10.1126/science.1123933

[31] Daniel C , Sartory NA , Zahn N , Radeke HH , Stein JM . Immune modulatory treatment of trinitrobenzene sulfonic acid colitis with calcitriol is associated with a change of a T helper (Th) 1/Th17 to a Th2 and regulatory T cell profile. J Pharmacol Exp Therap. 2008;324(1):23‐33. 17911375 10.1124/jpet.107.127209

[32] Sly LM , Lopez M , Nauseef WM , Reiner NE . 1α, 25‐Dihydroxyvitamin D3‐induced monocyte antimycobacterial activity is regulated by phosphatidylinositol 3‐kinase and mediated by the NADPH‐dependent phagocyte oxidase. J Biol Chem. 2001;276(38):35482‐35493. 11461902 10.1074/jbc.M102876200

[33] Cantorna MT , Snyder L , Lin YD , Yang L . Vitamin D and 1, 25 (OH) 2D regulation of T cells. Nutrients. 2015;7(4):3011‐3021. 25912039 10.3390/nu7043011 PMC4425186

[34] Jeffery LE , Burke F , Mura M , et al. 1, 25‐Dihydroxyvitamin D3 and IL‐2 combine to inhibit T cell production of inflammatory cytokines and promote the development of regulatory T cells expressing CTLA‐4 and FoxP3. J Immunol. 2009;183(9):5458‐5467. 19843932 10.4049/jimmunol.0803217 PMC2810518

[35] Aranow C . Vitamin D and the immune system. J Invest Med. 2011;59(6):881‐886. 10.231/JIM.0b013e31821b8755 PMC3166406 21527855

[36] Lei GS , Zhang C , Cheng BH , Lee CH . Mechanisms of action of vitamin D as supplemental therapy for pneumocystis pneumonia. Antimicrob Agents Chemother. 2017;61(10):e01226‐17. 28760906 10.1128/AAC.01226-17 PMC5610499

[37] Mousavi S , Bereswill S , Heimesaat MM . Immunomodulatory and antimicrobial effects of vitamin C. Eur J Microbio Immunol. 2019;9(3):73‐79. 10.1556/1886.2019.00016 PMC6798581 31662885

[38] Colunga Biancatelli RM , Berrill M , Marik PE . The antiviral properties of vitamin C. Exp Rev Anti‐Infective Therapy. 2020;18(2):99‐101. 10.1080/14787210.2020.1706483 31852327

[39] Penna G , Amuchastegui S , Giarratana N , et al. 1, 25‐Dihydroxyvitamin D3 selectively modulates tolerogenic properties in myeloid but not plasmacytoid dendritic cells. J Immunol. 2007;178(1):145‐153. 17182549 10.4049/jimmunol.178.1.145

[40] Széles L , Keresztes G , Töröcsik D , et al. 1, 25‐dihydroxyvitamin D3 is an autonomous regulator of the transcriptional changes leading to a tolerogenic dendritic cell phenotype. J Immunol. 2009;182(4):2074‐2083. 19201860 10.4049/jimmunol.0803345

[41] Chen Y , Zhang J , Ge X , Du J , Deb DK , Li YC . Vitamin D receptor inhibits nuclear factor κB activation by interacting with IκB kinase β protein. J Biol Chem. 2013;288(27):19450‐19458. 23671281 10.1074/jbc.M113.467670 PMC3707648

[42] Arboleda JF , Urcuqui‐Inchima S . Vitamin D supplementation: a potential approach for coronavirus/covid‐19 therapeutics. Front Immunol. 2020;11. 10.3389/fimmu.2020.01523 PMC7324720 32655583

[43] Beilfuss A , Sowa JP , Sydor S , et al. Vitamin D counteracts fibrogenic TGF‐β signalling in human hepatic stellate cells both receptor‐dependently and independently. Gut. 2015;64(5):791‐799. 25134788 10.1136/gutjnl-2014-307024

[44] Kong J , Zhu X , Shi Y , et al. VDR attenuates acute lung injury by blocking ang‐2‐Tie‐2 pathway and renin‐angiotensin system. Mol Endocrinol. 2013;27(12):2116‐2125. 24196349 10.1210/me.2013-1146 PMC3857197

[45] Gruber‐Bzura BM . Vitamin D and influenza—prevention or therapy. Int J Mol Sci. 2018;19(8):2419. 30115864 10.3390/ijms19082419 PMC6121423

[46] Singh S , Kola P , Kaur D , et al. Therapeutic Potential of Nutraceuticals and Dietary Supplements in the Prevention of Viral Diseases: A Review. Front Nutr. 2021;8:679312. 10.3389/fnut.2021.679312 PMC8484310 34604272

[47] Martineau AR , MacLaughlin BD , Hooper RL , et al. Double‐blind randomised placebo‐controlled trial of bolus‐dose vitamin D3 supplementation in adults with asthma (ViDiAs). Thorax. 2015;70:451‐457. 25724847 10.1136/thoraxjnl-2014-206449

[48] Zdrenghea MT , Makrinioti H , Bagacean C , Bush A , Johnston SL , Stanciu LA . Vitamin D modulation of innate immune responses to respiratory viral infections. Rev Med Virol. 2017;27(1):e1909. 10.1002/rmv.1909 27714929

[49] Lips P , Cashman KD , Lamberg‐Allardt C , et al. Current vitamin D status in European and Middle East countries and strategies to prevent vitamin D deficiency: a position statement of the European Calcified Tissue Society. European Journal of Endocrinology. 2019;180(4):P23–P54. 30721133 10.1530/EJE-18-0736

[50] Vankadari N , Wilce JA . Emerging COVID‐19 coronavirus: glycan shield and structure prediction of spike glycoprotein and its interaction with human CD26. Emerg Microbes Infect. 2020;9(1):601‐604. 32178593 10.1080/22221751.2020.1739565 PMC7103712

[51] Meltzer DO , Best TJ , Zhang H , Vokes T , Arora V , Solway J . Association of vitamin D deficiency and treatment with COVID‐19 incidence. Med Rxiv. 2020.

[52] Castillo ME , Costa LM , Barrios JM , et al. Effect of calcifediol treatment and best available therapy versus best available therapy on intensive care unit admission and mortality among patients hospitalized for COVID‐19: a pilot randomized clinical study. J Steroid Biochem Mol Biol. 2020;203:105751. 32871238 10.1016/j.jsbmb.2020.105751 PMC7456194

[53] Allegra A , Tonacci A , Pioggia G , Musolino C , Gangemi S . Vitamin deficiency as risk factor for SARS‐CoV‐2 infection: correlation with susceptibility and prognosis. Eur Rev Med Pharmacol Sci. 2020;24(18):9721‐9738. 33015818 10.26355/eurrev_202009_23064

[54] Panagiotou G , Tee SA , Ihsan Y , et al. Low serum 25‐hydroxyvitamin D (25 [OH] D) levels in patients hospitalized with COVID‐19 are associated with greater disease severity. Clin Endocrinol (Oxf). 2020;93(4):508‐511. 32621392 10.1111/cen.14276 PMC7361912

[55] Baktash V , Hosack T , Patel N . Vitamin D status and outcomes for hospitalized older patients with COVID‐19. Postgrad Med J. 2020;97(1149):442‐447. 32855214 10.1136/postgradmedj-2020-138712 PMC7456620

[56] Mardani R , Alamdary A , Nasab SM , Gholami R , Ahmadi N , Gholami A . Association of vitamin D with the modulation of the disease severity in COVID‐19. Virus Res. 2020;289:198148. 32866536 10.1016/j.virusres.2020.198148 PMC7455115

[57] Merzon E , Tworowski D , Gorohovski A , et al. Low plasma 25 (OH) vitamin D level is associated with increased risk of COVID‐19 infection: an Israeli population‐based study. FEBS J. 2020;287(17):3693‐3702. 32700398 10.1111/febs.15495 PMC7404739

[58] Rhodes JM , Subramanian S , Laird E , Griffin G , Kenny RA . Perspective: vitamin D deficiency and COVID‐19 severity–plausibly linked by latitude, ethnicity, impacts on cytokines, ACE2 and thrombosis. J Intern Med. 2021;289(1):97‐115. 32613681 10.1111/joim.13149 PMC7361294

[59] Ohaegbulam KC , Swalih M , Patel P , Smith MA , Perrin R . Vitamin D supplementation in COVID‐19 patients: a clinical case series. Amer J Therapeutics. 2020;e485. 10.1097/MJT.0000000000001222 PMC7473790 32804682

[60] Quesada‐Gomez JM , Entrenas‐Castillo M , Bouillon R . Vitamin D receptor stimulation to reduce acute respiratory distress syndrome (ARDS) in patients with coronavirus SARS‐CoV‐2 infections: revised Ms SBMB 2020_166. J Steroid Biochem Mol Biol. 2020;202:105719. 32535032 10.1016/j.jsbmb.2020.105719 PMC7289092

[61] Amrein K , Scherkl M , Hoffmann M , et al. Vitamin D deficiency 2.0: an update on the current status worldwide. Euro J Clinic Nutr. 2020;74(11):1498‐1513. 10.1038/s41430-020-0558-y PMC7091696 31959942

[62] Alipio, M. Vitamin D supplementation could possibly improve clinical outcomes of patients infected with Coronavirus‐2019 (COVID‐19). 2020.

[63] Wu S , Sun J . Vitamin D, vitamin D receptor, and macroautophagy in inflammation and infection. Discov Med. 2011;11(59):325. 21524386 PMC3285235

[64] Høyer‐Hansen M , Nordbrandt SP , Jäättelä M . Autophagy as a basis for the health‐promoting effects of vitamin D. Trends Mol Med. 2010;16(7):295‐302. 20488750 10.1016/j.molmed.2010.04.005

[65] Hong CW . Current understanding in neutrophil differentiation and heterogeneity. Immune Net. 2017;17(5):298. 10.4110/in.2017.17.5.298 PMC5662779 29093651

[66] Lescure FX , Bouadma L , Nguyen D , et al. Clinical and virological data of the first cases of COVID‐19 in Europe: a case series. Lancet Infect Dis. 2020;20(6):697‐706. 32224310 10.1016/S1473-3099(20)30200-0 PMC7156120

[67] Marik PE , Kory P , Varon J . Does vitamin D status impact mortality from SARS‐CoV‐2 infection. Med Drug Discov. 2020;6:100041. 32352080 10.1016/j.medidd.2020.100041 PMC7189189

[68] Martens PJ , Gysemans C , Verstuyf A , Mathieu C . Vitamin D's effect on immune function. Nutrients. 2020;12(5):1248. 32353972 10.3390/nu12051248 PMC7281985

[69] Christakos S , Hewison M , Gardner DG , et al. Vitamin D: beyond bone. Ann N Y Acad Sci. 2013;1287(1):45. 23682710 10.1111/nyas.12129 PMC3717170

[70] Khalil I , Barma P . Sub‐continental atmosphere and inherent immune system may have impact on novel Corona Virus' 2019 (nCovid‐19) prevalence in South East Asia. Mymensingh Med J. 2020;29(2):473‐480. 32506109

[71] Benskin LL . A basic review of the preliminary evidence that COVID‐19 risk and severity is increased in vitamin D deficiency. Front Public Health. 2020;8:513. 33014983 10.3389/fpubh.2020.00513 PMC7513835

[72] Aloia JF , Li‐Ng M . RE: epidemic Influenza and Vitamin D. Epidemiol Infect. 2007;135(7):1095‐1098. 17352842 10.1017/S0950268807008308 PMC2870688

[73] Hoan NX , Van Tong H , Le Huu Song CG , Velavan TP . Vitamin D deficiency and hepatitis viruses‐associated liver diseases: a literature review. World J Gastroenterol. 2018;24(4):445. 29398866 10.3748/wjg.v24.i4.445 PMC5787780

[74] Zhou J , Du J , Huang L , Wang Y , Shi Y , Lin H . Preventive effects of vitamin D on seasonal influenza a in infants: a multicenter, randomized, open, controlled clinical trial. Pediatr Infect Dis J. 2018;37(8):749‐754. 29315160 10.1097/INF.0000000000001890

[75] Urashima M , Segawa T , Okazaki M , Kurihara M , Wada Y , Ida H . Randomized trial of vitamin D supplementation to prevent seasonal influenza a in schoolchildren. Am J Clin Nutr. 2010;91(5):1255‐1260. 20219962 10.3945/ajcn.2009.29094

[76] Malek Mahdavi A . A brief review of interplay between vitamin D and angiotensin‐converting enzyme 2: Implications for a potential treatment for COVID‐19. Rev Med Virol. 2020;30(5):e2119. 32584474 10.1002/rmv.2119 PMC7362103

[77] Iddir M , Brito A , Dingeo G , et al. Strengthening the immune system and reducing inflammation and oxidative stress through diet and nutrition: considerations during the COVID‐19 crisis. Nutrients. 2020;12(6):1562. 32471251 10.3390/nu12061562 PMC7352291

[78] Tsujino I , Ushikoshi‐Nakayama R , Yamazaki T , Matsumoto N , Saito I . Pulmonary activation of vitamin D3 and preventive effect against interstitial pneumonia. J Clinic Biochem Nut. 2019;65(3):245‐251. 10.3164/jcbn.19-48 PMC6877402 31777427

[79] Martineau AR . Vitamin D supplementation and musculoskeletal health. Lancet Diabetes Endocrinol. 2019;7(2):86‐87. 30683218 10.1016/S2213-8587(18)30349-8

[80] Tan CW , Ho LP , Kalimuddin S , et al. A cohort study to evaluate the effect of combination vitamin D, magnesium and vitamin B12 (DMB) on progression to severe outcome in older COVID‐19 patients. Med Rxiv. 2020.

[81] Glicio, EJ . Vitamin D level of mild and severe elderly cases of COVID‐19: a preliminary report. 2020.

[82] Daneshkhah A , Agrawal V , Eshein A , et al. The possible role of vitamin D in suppressing cytokine storm and associated mortality in COVID‐19 patients [preprint]. Infect Dis. 2020.

[83] Raharusun, P. ; Priambada, S. ; Budiarti, C. ; Agung, E. ; Budi, C. Patterns of COVID‐19 mortality and vitamin D: an Indonesian study. 2020.

[84] Li Y , Li Q , Zhang N , Liu Z . Sunlight and vitamin D in the prevention of coronavirus disease (COVID‐19) infection and mortality in the United States. Sci Rep. 2020.

[85] Martineau AR , Jolliffe DA , Hooper RL , et al. Vitamin D supplementation to prevent acute respiratory tract infections: systematic review and meta‐analysis of individual participant data. BMJ. 2017;356:i6583. 28202713 10.1136/bmj.i6583 PMC5310969

[86] Vieth R , Kimball S , Hu A , Walfish PG . Randomized comparison of the effects of the vitamin D 3 adequate intake versus 100 mcg (4000 IU) per day on biochemical responses and the wellbeing of patients. Nutr J. 2004;3(1):1. 15260882 10.1186/1475-2891-3-8 PMC506781

[87] Bischoff‐Ferrari HA , Shao A , Dawson‐Hughes B , Hathcock J , Giovannucci E , Willett WC . Benefit–risk assessment of vitamin D supplementation. Osteopor Int. 2010;21(7):1121‐1132. 10.1007/s00198-009-1119-3 PMC3062161 19957164

[88] Giménez VMM , Sanz RL , Marón FJM , Ferder L , Manucha W . Vitamin D‐RAAS connection: an integrative standpoint into cardiovascular and neuroinflammatory disorders. Curr Protein Pept Sci. 2020;21:948‐954. 32504501 10.2174/1389203721666200606220719

[89] Xu J , Yang J , Chen J , Luo Q , Zhang Q , Zhang H . Vitamin D alleviates lipopolysaccharide‐induced acute lung injury via regulation of the renin‐angiotensin system. Mol Med Rep. 2017;16:7432‐7438. 28944831 10.3892/mmr.2017.7546 PMC5865875

[90] Ambrosino I , Barbagelata E , Ortona E , et al. Gender differences in patients with COVID‐19: a narrative review. Monaldi Arch Chest Dis. 2020;90. 10.4081/monaldi.2020.1389 32449614

[91] Cai H . Sex difference and smoking predisposition in patients with COVID‐19. Lancet Respir Med. 2020;8:e20. 32171067 10.1016/S2213-2600(20)30117-X PMC7103991

[92] Dalpiaz PL , Lamas AZ , Caliman IF , et al. Sex hormones promote opposite effects on ACE and ACE2 activity, hypertrophy and cardiac contractility in spontaneously hypertensive rats. PLoS ONE. 2015;10:e0127515. 26010093 10.1371/journal.pone.0127515 PMC4444272

[93] Samidurai A , Das A . Cardiovascular complications associated with COVID‐19 and potential therapeutic strategies. Int J Mol Sci. 2020;21:6790. 32947927 10.3390/ijms21186790 PMC7554795

[94] Richardson S , Hirsch JS , Narasimhan M , et al. Presenting characteristics, comorbidities, and outcomes among 5700 patients hospitalized with COVID‐19 in the New York City area. JAMA. 2020;323:2052‐2059. 32320003 10.1001/jama.2020.6775 PMC7177629

[95] Jin JM , Bai P , He W , et al. Gender differences in patients with COVID‐19: focus on severity and mortality. Front Public Health. 2020;8:152. 32411652 10.3389/fpubh.2020.00152 PMC7201103

[96] Stokes EK , Zambrano LD , Anderson KN , et al. Coronavirus disease 2019 case surveillance–United States, January 22–may 30, 2020. MMWR Morb Mortal Wkly Rep. 2020;69(24):759‐765. 32555134 10.15585/mmwr.mm6924e2 PMC7302472

[97] Muscogiuri G , Barrea L , Somma CD , et al. Sex differences of vitamin D status across BMI classes: an observational prospective cohort study. Nutrients. 2019;11:3034. 31842281 10.3390/nu11123034 PMC6950363

[98] Spanier JA , Nashold FE , Mayne CG , Nelson CD , Hayes CE . Vitamin D and estrogen synergy in Vdr‐expressing CD4(+) T cells is essential to induce Helios(+)FoxP3(+) T cells and prevent autoimmune demyelinating disease. J Neuroimmunol. 2015;286:48‐58. 26298324 10.1016/j.jneuroim.2015.06.015

[99] Mukherjee S , Pahan K . Is COVID‐19 gender‐sensitive? J Neuroimmune Pharmacol. 2021;16:38‐47. 33405098 10.1007/s11481-020-09974-z PMC7786186

[100] Silberstein M . COVID‐19 and IL‐6: why vitamin D (probably) helps but tocilizumab might not. Eur J Pharmacol. 2021;899:174031. 33722593 10.1016/j.ejphar.2021.174031 PMC7954769

[101] Song Y , Qayyum S , Greer RA , et al. Vitamin D3 and its hydroxyderivatives as promising drugs against covid‐19: a computational study. J Biomol Struct Dyn. 2021;1‐17. 10.1080/07391102.2021.1964601 PMC8858339 34415218

[102] Glinsky GV . Tripartite combination of candidate pandemic mitigation agents: vitamin D, quercetin, and estradiol manifest properties of medicinal agents for targeted mitigation of the COVID‐19 pandemic defined by genomics‐guided tracing of SARS‐CoV‐2 targets in human cells. Biomedicine. 2020;8:129. 10.3390/biomedicines8050129 PMC7277789 32455629

[103] Whittemore PB . COVID‐19 fatalities, latitude, sunlight, and vitamin D. Am J Infect Control. 2020;48:1042‐1044. 32599103 10.1016/j.ajic.2020.06.193 PMC7319635

[104] Baktash V , Hosack T , Patel N , et al. Vitamin D status and outcomes for hospitalised older patients with COVID‐19. Postgrad Med J. 2021;97:442‐447. 32855214 10.1136/postgradmedj-2020-138712 PMC7456620

[105] Meltzer DO , Best TJ , Zhang H , Vokes T , Arora V , Solway J . Association of vitamin D status and other clinical characteristics with COVID‐19 test results. JAMA Netw Open. 2020;3:e2019722. 32880651 10.1001/jamanetworkopen.2020.19722 PMC7489852

[106] Radujkovic A , Hippchen T , Tiwari‐Heckler S , Dreher S , Boxberger M , Merle U . Vitamin D deficiency and outcome of COVID‐19 patients. Nutrients. 2020;12:2757. 32927735 10.3390/nu12092757 PMC7551780

[107] Lakkireddy M , Gadiga SG , Malathi RD , et al. Impact of daily high dose oral vitamin D therapy on the inflammatory markers in patients with COVID 19 disease. Sci Rep. 2021;11:10641. 34017029 10.1038/s41598-021-90189-4 PMC8138022

[108] Ünsal YA , Gül ÖÖ , Cander S , et al. Retrospective analysis of vitamin D status on ınflammatory markers and course of the disease in patients with COVID‐19 infection. J Endocrinol Invest. 2021;44(12):2601‐2607. 33818731 10.1007/s40618-021-01566-9 PMC8020370

[109] Murai IH , Fernandes AL , Sales LP , et al. Effect of a single high dose of vitamin D3 on hospital length of stay in patients with moderate to severe COVID‐19: a randomized clinical trial. JAMA. 2021;325:1053‐1060. 33595634 10.1001/jama.2020.26848 PMC7890452

[110] Rastogi A , Bhansali A , Khare N , et al. Short term, high‐dose vitamin D supplementation for COVID‐19 disease: a randomised, placebo‐controlled, study (SHADE study). Postgrad Med J. 2020;98:87‐90. 33184146 10.1136/postgradmedj-2020-139065

[111] Annweiler C , Hanotte B , l'Eprevier CGD , Sabatier JM , Lafaie L , Célarier T . Vitamin D and survival in COVID‐19 patients: a quasi‐experimental study. J Steroid Biochem Mol Biol. 2020;204:105771. 33065275 10.1016/j.jsbmb.2020.105771 PMC7553119

[112] Munshi R , Hussein MH , Toraih EA , et al. Vitamin D insufficiency as a potential culprit in critical COVID‐19 patients. J Med Virol. 2021;93:733‐740. 32716073 10.1002/jmv.26360

[113] Stroehlein JK , Wallqvist J , Iannizzi C , et al. Vitamin D supplementation for the treatment of COVID‐19: a living systematic review. Cochrane Database Syst Rev. 2021;5:Cd015043. 34029377 10.1002/14651858.CD015043 PMC8406457

[114] Annweiler C , Beaudenon M , Gautier J , et al. COvid‐19 and high‐dose VITamin D supplementation TRIAL in high‐risk older patients (COVIT‐TRIAL): study protocol for a randomized controlled trial. Trials. 2020;21:1031. 33371905 10.1186/s13063-020-04928-5 PMC7768266

[115] Annweiler G , Corvaisier M , Gautier J , et al. Vitamin D supplementation associated to better survival in hospitalized frail elderly COVID‐19 patients: the GERIA‐COVID quasi‐experimental study. Nutrients. 2020;12:3377. 33147894 10.3390/nu12113377 PMC7693938

[116] Pereira M , Damascena AD , Azevedo LMG , Oliveira TDA , Santana JDM . Vitamin D deficiency aggravates COVID‐19: systematic review and meta‐analysis. Crit Rev Food Sci Nutr. 2020;62(5):1‐9. 33146028 10.1080/10408398.2020.1841090

[117] Liu Y , Yan LM , Wan L , et al. Viral dynamics in mild and severe cases of COVID‐19. Lancet Infect Dis. 2020;20:656‐657. 32199493 10.1016/S1473-3099(20)30232-2 PMC7158902

[118] Tan CW , Ho LP , Kalimuddin S , et al. Cohort study to evaluate the effect of vitamin D, magnesium, and vitamin B(12) in combination on progression to severe outcomes in older patients with coronavirus (COVID‐19). Nutrition. 2020;79:111017. 33039952 10.1016/j.nut.2020.111017 PMC7832811

[119] Meltzer DO , Best TJ , Zhang H , Vokes T , Arora V , Solway J . Association of Vitamin D Deficiency and Treatment with COVID‐19 incidence. MedRxiv. 2020.

[120] Kaufman HW , Niles JK , Kroll MH , Bi C , Holick MF . SARS‐CoV‐2 positivity rates associated with circulating 25‐ hydroxyvitamin D levels. PLoS One. 2020;15:e0239252. 32941512 10.1371/journal.pone.0239252 PMC7498100

[121] Jain A , Chaurasia R , Sengar NS , Singh M , Mahor S , Narain S . Analysis of vitamin D level among asymptomatic and critically ill COVID‐19 patients and its correlation with inflammatory markers. Sci Rep. 2020;10:20191. 33214648 10.1038/s41598-020-77093-z PMC7677378

[122] Carpagnano GE , Lecce VD , Quaranta VN , et al. Vitamin D deficiency as a predictor of poor prognosis in patients with acute respiratory failure due to COVID‐19. J Endocrinol Invest. 2021;44:765‐771. 32772324 10.1007/s40618-020-01370-x PMC7415009

[123] Padhi S , Suvankar S , Panda VK , Pati A , Panda AK . Lower levels of vitamin D are associated with SARS‐CoV‐2 infection and mortality in the Indian population: an observational study. Int Immunopharmacol. 2020;88:107001. 33182040 10.1016/j.intimp.2020.107001 PMC7489890

[124] Ling SF , Broad E , Murphy R , et al. High‐dose cholecalciferol booster therapy is associated with a reduced risk of mortality in patients with COVID‐19: a cross‐sectional multi‐Centre observational study. Nutrients. 2020;12:3799. 33322317 10.3390/nu12123799 PMC7763301

[125] Smet DD , Smet KD , Herroelen P , Gryspeerdt S , Martens GA . Serum 25(OH)D level on hospital admission associated with COVID‐19 stage and mortality. Am J Clin Pathol. 2021;155:381‐388. 33236114 10.1093/ajcp/aqaa252 PMC7717135

[126] Karahan S , Katkat F . Impact of serum 25(OH) vitamin D level on mortality in patients with COVID‐19 in Turkey. J Nutr Health Aging. 2021;25:189‐196. 33491033 10.1007/s12603-020-1479-0 PMC7533663

[127] AlSafar H , Grant WB , Hijazi R , et al. COVID‐19 disease severity and death in relation to vitamin D status among SARS‐CoV‐2‐positive UAE residents. Nutrients. 1714;2021:13. 10.3390/nu13051714 PMC8159141 34069412

[128] Orchard L , Baldry M , Nasim‐Mohi M , et al. Vitamin‐D levels and intensive care unit outcomes of a cohort of critically ill COVID‐19 patients. Clin Chem Lab Med. 2021;59:1155‐1163. 33554566 10.1515/cclm-2020-1567

[129] Osman W , Fahdi FA , Salmi IA , Khalili HA , Gokhale A , Khamis F . Serum calcium and vitamin D levels: correlation with severity of COVID‐19 in hospitalized patients in Royal Hospital. Oman Int J Infect Dis. 2021;107:153‐163. 33892191 10.1016/j.ijid.2021.04.050 PMC8057687

[130] Diaz‐Curiel M , Cabello A , Arboiro‐Pinel R , et al. The relationship between 25(OH) vitamin D levels and COVID‐19 onset and disease course in Spanish patients. J Steroid Biochem Mol Biol. 2021;212:105928. 34091026 10.1016/j.jsbmb.2021.105928 PMC8180342

[131] Angelidi AM , Belanger MJ , Lorinsky MK , et al. Vitamin D status is associated with In‐hospital mortality and mechanical ventilation: a cohort of COVID‐19 hospitalized patients. Mayo Clin Proc. 2021;96:875‐886. 33714594 10.1016/j.mayocp.2021.01.001 PMC7834253

[132] Jevalikar G , Mithal A , Singh A , et al. Lack of association of baseline 25‐hydroxyvitamin D levels with disease severity and mortality in Indian patients hospitalized for COVID‐19. Sci Rep. 2021;11:6258. 33737631 10.1038/s41598-021-85809-y PMC7973709

[133] Alcala‐Diaz JF , Limia‐Perez L , Gomez‐Huelgas R , et al. Calcifediol treatment and hospital mortality due to COVID‐19: a cohort study. Nutrients. 1760;2021:13. 10.3390/nu13061760 PMC8224356 34064175

[134] Cangiano B , Fatti LM , Danesi L , et al. Mortality in an Italian nursing home during COVID‐19 pandemic: correlation with gender, age, ADL, vitamin D supplementation, and limitations of the diagnostic tests. Aging. 2020;12:24522‐24534. 33353888 10.18632/aging.202307 PMC7803543

[135] Fasano A , Cereda E , Barichella M , et al. COVID‐19 in Parkinson's disease patients living in Lombardy. Italy Mov Disord. 2020;35:1089‐1093. 32484584 10.1002/mds.28176 PMC7300944

[136] Giannini S , Passeri G , Tripepi G , et al. Effectiveness of In‐hospital cholecalciferol use on clinical outcomes in comorbid COVID‐19 patients: a hypothesis‐generating study. Nutrients. 2021;13:219. 33466642 10.3390/nu13010219 PMC7828675

[137] Israel A , Schäffer AA , Cicurel A , et al. Identification of drugs associated with reduced severity of COVID‐19–a case‐control study in a large population. Elife. 2021;10:e68165. 34313216 10.7554/eLife.68165 PMC8321549

[138] Loucera C , Peña‐Chilet M , Esteban‐Medina M , et al. Real world evidence of calcifediol use and mortality rate of COVID‐19 hospitalized in a large cohort of 16,401 Andalusian patients. MedRxiv. 2021. 10.1038/s41598-021-02701-5 PMC8642445 34862422

[139] Sabico S , Enani MA , Sheshah E , et al. Effects of a 2‐week 5000 IU versus 1000 IU vitamin D3 supplementation on recovery of symptoms in patients with mild to moderate Covid‐19: a randomized clinical trial. Nutrients. 2021;13:2170. 34202578 10.3390/nu13072170 PMC8308273

[140] Oristrell J , Oliva JC , Casado E , et al. Vitamin D supplementation and COVID‐19 risk: a population‐based, cohort study. J Endocrinol Invest. 2021;45(1):1‐13. 34273098 10.1007/s40618-021-01639-9 PMC8285728

[141] Nogues X , Ovejero D , Pineda‐Moncusí M , et al. Calcifediol treatment and COVID‐19‐related outcomes. J Clin Endocrinol Metab. 2021;106(10):e4017‐e4027. 34097036 10.1210/clinem/dgab405 PMC8344647

[142] Ma H , Zhou T , Heianza Y , Qi L . Habitual use of vitamin D supplements and risk of coronavirus disease 2019 (COVID‐19) infection: a prospective study in UK biobank. Am J Clin Nutr. 2021;113:1275‐1281. 33515005 10.1093/ajcn/nqaa381 PMC7929381

[143] D'Avolio A , Avataneo V , Manca A , et al. 25‐hydroxyvitamin D concentrations are lower in patients with positive PCR for SARS‐CoV‐2. Nutrients. 2020;12:1359. 32397511 10.3390/nu12051359 PMC7285131

[144] Fasano A , Cereda E , Barichella M , et al. COVID‐19 in Parkinson's disease patients living in Lombardy, Italy. Mov Disord. 2020;35:1089‐1093. 32484584 10.1002/mds.28176 PMC7300944

[145] NICE . Vitamin D for covid‐19: evidence reviews for the use of vitamin D supplementation as prevention and treatment of covid‐19. 2020. 33378142

[146] Fabbri A , Infante M , Ricordi C . Editorial‐vitamin D status: a key modulator of innate immunity and natural defense from acute viral respiratory infections. Eur Rev MedPharmacol Sci. 2020;24(7):4048‐4052. 10.26355/eurrev_202004_20876 32329882

[147] Santos RN , Maeda SS , Jardim JR , Lazaretti‐Castro M . Reasons to avoid vitamin D deficiency during COVID‐19 pandemic. Archives Endocrinol Metabol. 2020;64(5):498‐506. 10.20945/2359-3997000000291 PMC10118971 34033288

[148] Cipriani C , Romagnoli E , Pepe J , et al. Long‐term bioavailability after a single oral or intramuscular administration of 600,000 IU of ergocalciferol or cholecalciferol: implications for treatment and prophylaxis. J Clin Endocrinol Metab. 2013;98(7):2709‐2715. 23766519 10.1210/jc.2013-1586

[149] Martineau AR , Jolliffe DA , Greenberg L , et al. Vitamin D supplementation to prevent acute respiratory infections: individual participant data meta‐analysis. Health Tech Assessment. 2019;23(2):1‐44. 10.3310/hta23020 PMC6369419 30675873

[150] Marcinowska‐Suchowierska E , Kupisz‐Urbańska M , Łukaszkiewicz J , Płudowski P , Jones G . Vitamin D toxicity–a clinical perspective. Front Endocrinol. 2018;9:550. 10.3389/fendo.2018.00550 PMC6158375 30294301

[151] Liu G , Hong T , Yang J . A single large dose of vitamin D could be used as a means of coronavirus disease 2019 prevention and treatment. Drug Des Devel Ther. 2020;14:3429. 10.2147/DDDT.S271754 PMC7457388 32904593

[152] Hastie CE , Mackay DF , Ho F , et al. Vitamin D concentrations and COVID‐19 infection in UK biobank. Diabetes Metab Syndr Clin Res Rev. 2020;14:561‐565. 10.1016/j.dsx.2020.04.050 PMC7204679 32413819

[153] Weir EK , Thenappan T , Bhargava M , Chen Y . Does vitamin D deficiency increase the severity of COVID‐19? Clin Med. 2020;20:e107‐e108. 10.7861/clinmed.2020-0301 PMC7385774 32503801

[154] Martineau AR , Jolliffe DA , Hooper RL , et al. Vitamin D supplementation to prevent acute respiratory tract infections: systematic review and meta‐analysis of individual participant data. BMJ. 2019;356:2917. 10.1136/bmj.i6583 PMC5310969 28202713

[155] Daneshkhah A , Agrawal V , Eshein A et al. The possible role of vitamin D in suppressing cytokine storm and associated mortality in COVID‐19 patients, 2020, medRxiv preprint.

[156] Grigoriou EV , Trovas G , Papaioannou N . Serum 25‐ hydroxyvitamin D status, quantitative ultrasound parameters, and their determinants in Greek population. Arch Osteoporos. 2018;13(1):111. 30324335 10.1007/s11657-018-0526-5

[157] Rhodes JS , Laird E , Griffin G , Kenny RA . Perspective: vitamin D deficiency and COVID‐19 severity ‐ plausibly linked by latitude, ethnicity, impacts on cytokines, ACE2, and thrombosis (R1). J Intern Med. 2020;2:97‐115. 10.1111/joim.13149 PMC7361294 32613681

[158] Kohlmeier M . Avoidance of vitamin D deficiency to slow the COVID‐19 pandemic. BMJ Nutr Prev Health. 2020;3:67‐73. 10.1136/bmjnph-2020-000096 PMC7295862 33230496

